# Study of the Kinetics of the Determinants of Performance During a Mountain Ultramarathon: Multidisciplinary Protocol of the First Trail Scientifique de Clécy 2021

**DOI:** 10.2196/38027

**Published:** 2022-06-15

**Authors:** Benoit Mauvieux, Corentin Hingrand, Joffrey Drigny, Amir Hodzic, Pauline Baron, Rémy Hurdiel, Romain Jouffroy, Jean-Charles Vauthier, Mathias Pessiglione, Antonius Wiehler, Francis Degache, Sébastien Pavailler, Elsa Heyman, Mathilde Plard, Philippe Noirez, Blaise Dubois, Jean François Esculier, Anh Phong Nguyen, Joachim Van Cant, Olivier Roy Baillargeon, Benoit Pairot de Fontenay, Pierre Louis Delaunay, Stéphane Besnard

**Affiliations:** 1 U1075 Comete/INSERM Université de Caen Caen France; 2 Unité de Médecine du Sport Centre Hospitalier Universitaire de Caen Normandie Caen France; 3 ULR 7369 - Unité de Recherche Pluridisciplinaire Sport, Santé, Société Université du Littoral Côte d'Opale Dunkerque France; 4 Intensive Care Unit, Anaethesiology, SAMU, Necker Enfants Malades Hospital Assistance Publique - Hôpitaux de Paris Paris France; 5 IRMES - Institute for Research in Medicine and Epidemiology of Sport Institut National du Sport, de l'Expertise et de la Performance Paris France; 6 INSERM U-1018, Centre de recherche en Epidémiologie et Santé des Populations Paris Saclay University Paris France; 7 Departement de Medecine Générale Faculté de Médecine - Département du Grand Est de recherche en soins primaires Université de Lorraine Nancy France; 8 Motivation, Brain and Behavior lab Institut du cerveau et de la moelle épinière Inserm U1127, CNRS U9225 Université Pierre et Marie Curie (UPMC-Paris 6) Paris France; 9 MotionLab Le Mont sur Lausanne Switzerland; 10 Salomon Sport Science Laboratory Salomon SAS Annecy France; 11 ULR 7369 - Unité de Recherche Pluridisciplinaire Sport, Santé, Société Université de Lille LILLE France; 12 Institut Universitaire de France Paris France; 13 Espace et Sociétés UMR 6590 CNRS Université d'Angers Angers France; 14 Performance Santé Métrologie Société (EA7507) Université Reims Champagne Ardenne Reims France; 15 La Clinique du Coureur Lac Beauport, QC Canada; 16 Neuromusculoskeletal Laboratory Institut de Recherche Expérimentale et Clinique Catholic University of Louvain Louvain La Neuve Belgium; 17 Department of Physical Therapy Institut Parnasse-ISEI Brussels Belgium; 18 Inter-University Laboratory of Human Movement Biology Lyon 1 University Lyon France; 19 Explorations Fonctionnelles Neurologiques Centre Hospitalier Universitaire de Caen Caen France

**Keywords:** ultramarathon, trail-running, sports physiology, sleep deprivation, fatigue, blood biology, muscular function, biomechanics, motivation, cognition, self-esteem

## Abstract

**Background:**

The growing interest of the scientific community in trail running has highlighted the acute effects of practice at the time of these races on isolated aspects of physiological and structural systems; biological, physiological, cognitive, and muscular functions; and the psychological state of athletes. However, no integrative study has been conducted under these conditions with so many participants and monitoring of pre-, per-, and postrace variables for up to 10 days over a distance close to 100 miles.

**Objective:**

The aim of this study was to evaluate the kinetics of the performance parameters during a 156 km trail run and 6000 m of elevation gain in pre-, per-, and postrace conditions. The general hypothesis is based on significant alterations in the psychological, physiological, mechanical, biological, and cognitive parameters.

**Methods:**

The Trail Scientifique de Clécy took place on November 11, 2021. This prospective experimental study provides a comprehensive exploration of the constraints and adaptations of psychophysiological and sociological variables assessed in real race conditions during a trail running of 156 km on hilly ground and 6000 m of elevation gain (D+). The study protocol allowed for repeatability of study measurements under the same experimental conditions during the race, with the race being divided into 6 identical loops of 26 km and 1000 m D+. Measurements were conducted the day before and the morning of the race, at the end of each lap, after a pit stop, and up to 10 days after the race. A total of 55 participants were included, 43 (78%) men and 12 (22%) women, who were experienced in ultra–trail-running events and with no contraindications to the practice of this sport.

**Results:**

The launch of the study was authorized on October 26, 2021, under the trial number 21-0166 after a favorable opinion from the Comité de Protection des Personnes Ouest III (21.09.61/SIRIPH 2G 21.01586.000009). Of the 55 runners enrolled, 41 (75%) completed the race and 14 (25%) dropped out for various reasons, including gastric problems, hypothermia, fatigue, and musculoskeletal injuries. All the measurements for each team were completed in full. The race times (ie, excluding the measurements) ranged from 17.8206 hours for the first runner to 35.9225 hours for the last runner. The average time to complete all measurements for each lap was 64 (SD 3) minutes.

**Conclusions:**

The Trail Scientifique de Clécy, by its protocol, allowed for a multidisciplinary approach to the discipline. This approach will allow for the explanation of the studied parameters in relation to each other and observation of the systems of dependence and independence. The initial results are expected in June 2022.

**International Registered Report Identifier (IRRID):**

RR1-10.2196/38027

## Introduction

### Background

Trail running is a sport discipline defined by the International Trail Running Association as a pedestrian competition taking place in a natural environment in a semi–self-sufficient or self-sufficient manner and with respect for sports ethics, fairness, solidarity, and the environment [1].

The practice of the ultratrail, the leader of ultraendurance events, shows an exponentially popular craze. In France, for example, since the first edition of the Ultra Trail du Mont Blanc, the number of finishers has risen from 67 in 2003 to 1685 in 2017, to 1556 in 2019, and to 1521 in 2021, and the number of registration requests has risen from 722 to 5575 [2]. In 2001, 150 trails were organized in France, and there were >2500 in 2015 [3]. A study conducted by the French Federation of Sports and Leisure Industries reported a niche of approximately 3.5 million trail runners out of the 17 million *runners* identified in France in 2017 [4].

### A Craze for Research Around the Disciplines of Ultraendurance

In parallel with this popular craze, ultraendurance and ultratrail in particular are areas of increasing interest to the scientific community. When looking at the number of publications that include the terms *ultratrail* or *ultraendurance* or *ultramarathon* indexed on PubMed, 650 references are found, with a significant increase in recent years (a total of 3 in 2000, a total of 20 in 2010, a total of 65 in 2015, and a total of 70 in 2020). However, the volume of publications remains low compared with other disciplines; for example, the terms *soccer* or *tennis* lead to 9974 and 8127 indexed articles, respectively.

This contrast is linked to not only the relative youth of the ultraendurance disciplines but also the lack of funding available for research in sports medicine and sports science in general [2]. Furthermore, most of the publications were observational studies. Less than 5% of the articles listed were randomized controlled trials, and <1% were systematic reviews or meta-analyses [2].

Ultraendurance disciplines and the mountain ultramarathon, in particular, offer a vast field of research in public health, basic sciences, and human sciences. The relative youth of the discipline and the material and logistical constraints of setting up in situ biomedical research during mountain ultramarathon races explain the low number of studies [3].

For example, when analyzing the studies published on PubMed from 2019 to 2021 with the words *ultramarathon* or *ultratrail*, out of the 171 articles published, only 23 (13.5%) studies were able to conduct a pre-, per-, and postrace protocol. Unfortunately, these studies often focus on one variable or field of study [5-26], particularly cardiac, renal, or psychomotivational functions. Moreover, of these 23 studies, 12 (52%) protocols were conducted on a treadmill [5,13,19,27], on roads or nontechnical trails [6,10,12,18,23,28], on short distances [16], or in an extreme environment [7].

In 2019, the study by Belinchón-deMiguel et al [24] took an integrative approach to performance by integrating physiological measurements such as anthropometry, heart rate (HR), blood pressure, oxygen saturation, muscle strength, and hydration; participants’ training parameters; nutritional parameters; and psychological parameters such as perceived stress and general mental health status. However, these measurements were only performed in the pre-postrace period, as they could not shed light on their kinetics. Other parameters were not studied, such as psychomotivational factors, thermoregulation, cardiac function, myotendinous activity, and inflammation. The health risks associated with the practice of this discipline have not been studied.

The lack of data found in the literature on these questions, which are linked to the difficulty of setting up scientific protocols during events and associated with the predominant place occupied by the trail discipline, gave rise to the project in Clécy, Normandy, France.

To respond to this context, a consortium comprising several local (Centre Hospitalier Universitaire de Caen and laboratory COMETE U1075 Unit), national, and international research teams proposed to set up a common protocol to understand the kinetics of the psychophysiological mechanisms that contribute to performance during an ultratrail race, as well as the social determinants. To this end, measurements will be taken before, during, and after a trail of 156 km with 55 volunteers and experienced runners. This scientific study is the first in its format, with 55 participants over a long race with a positive elevation gain (6000 m) and bringing together 11 research laboratories for measurements in pre-, per- (6 standardized fixed points), and postrace (10 days of follow-up) variables.

### Objective

The objective of this protocol was to study in situ the kinetics of the factors determining performance in an ultraendurance trail-running event. This main objective is broken down into several scientific disciplines, each of which includes subobjectives (Textbox 1).

**Objectives based on scientific disciplines.**

**Physiological exploration**

- To study the relationship between thermoregulatory capacity and performance
- To quantify the degradation of the muscular and biomechanical determinants of performance, muscular function, locomotor function, and static and dynamic postural functions
- To study the variability of cardiorespiratory parameters, including heart rate, heart rate variability, and respiratory rate
- To analyze acute adaptations in cardiac volume, myocardial contractility, and relaxation using transthoracic echocardiography
- To study the per-effort and posteffort variations of biological markers of inflammation and cardiac, renal, and neurological functions
- To evaluate the impact of the ultratrail on the runners’ glycemic balance
- To evaluate sleep before, during, and after an ultraendurance race

**Biomechanical exploration**

- To study the variation of the elastic and architectural properties of the gastrocnemius-Achilles tendon complex using ultrasound elastography
- To study the effects of fatigue on the biomechanics of trial running
- To study the relationship between shoe-related needs and the morphological, biomechanical, and sensory characteristics of the ultraendurance runner

**Psychocognitive and sociological exploration**

- To assess spatial cognition
- To determine the effect of exercise combined with sleep deprivation on response time, sustained attention, and sleepiness
- To study the psychological determinants of performance in an ultraendurance sport
- To study the link between the profile of confirmed or elite participants in ultraendurance and their ability to be attentive to self, others, and the world by mobilizing different indicators

**Environmental exploration**

- To study the evolution of air quality and its association with physiological parameters during an ultraendurance race

## Methods

### Recruitment

This experimental study included 55 volunteer participants, 43 (78%) men and 12 (22%) women, aged between 25 and 70 years. On the basis of the high range of abandonment (ie, 25% on an ultraendurance race), we estimated a cohort of finishers of 40 to 45 runners. Recruitment started in January 2021 until September 2021 with an announcement on the social networks of the Trail Scientifique de Clécy. If the runners met the inclusion criteria, they were invited to a videoconference exposing the entire protocol, with the consent letter being provided for reading. The runners were definitively included in the study on November 10, 2021, after a medical examination.

The medical examination was completed using electrocardiography and cardiac echography. If an anomaly was detected, the runner was excluded from the study.

A medical check-up was conducted 24 hours after the finish line or withdrawal and then 1 to 2 months after the race by teleconsultation.

### Inclusion and Exclusion Criteria

To be eligible, participants had to verify all the defined inclusion and exclusion criteria (Textbox 2).

**Inclusion and exclusion criteria.**

**Inclusion criteria**

- Experienced runners voluntarily participating in the Trail Scientifique de Clécy (156 km/6000 D+)
- Participants who had already completed 2 ultratrail races (+160 km and −160 km), at least one of them in the past 24 months; the participants had to justify their events and rankings
- Participants affiliated with a social security system or those who were a beneficiary of such a system
- Participants who could speak and read the French language
- Healthy volunteers aged 25 to 70 years
- Participants with the ability to physically participate in the ultraendurance race
- Participants with the ability to provide written consent for participation in the study
- Participants whose usual place of residence is +2 hours or –2 hours from the Greenwich meridian
- Participants with a medical certificate of no contraindication to the practice of ultratrail for <1 year

**Exclusion criteria**

- Participants with cardiac or extracardiac contraindications to intense physical activity
- Participants who had run a mountain ultramarathon (160 km) after September 2, 2021
- Pregnant or breastfeeding women
- Minor participants
- Participants included in another biomedical research protocol during this study
- Participants who refused to participate or who had the inability to access or read the newsletter
- Participants with a swallowing disorder
- Participants with a chronic transit disorder, including Crohn disease and digestive cancer
- Participants with magnetic resonance imaging scheduled within 48 hours of the race
- Participants with a medical history of pulmonary pathology; cardiac pathology; arterial hypertension; or significant inflammatory, renal, cardiac, or neurological disease observed during the inclusion visit
- All runners undergoing medical treatment
- Participants with recent muscular and orthopedic injuries, limiting running for <15 days
- Participants with a history of ankle joint surgery (eg, arthrodesis)
- Participants with joint stiffness corresponding to ranges of <15° dorsal flexion and 35° plantar flexion
- Participants with a history of foot or ankle surgery
- Participants with significant sensory disturbances in the foot
- Participants with pathological asymmetry between the right and left feet
- Participants with lower-limb pathology or trauma
- Participants with central and peripheral neurological pathologies
- Participants who experienced a time difference of >2 hours in the month preceding the event (jet lag)

### Selection of Variables Identified as Determinants of Performance

The selected variables were based on scientific work in an ultramarathon, trail running, or ultratrail (Table 1).

**Table 1:** Parameters selected for our study, which have been reviewed in the literature.

Parameters		Studies
**Muscle function**		
	Muscle strength and power	[25,26,29]
	Neuromuscular fatigue	[30]
**Thermoregulation**		
	Core temperature	[31,32]
	Skin temperature	[31,32]
	Regulation	[33]
**Cardiac function**		
	Blood pressure	[11,21]
	Ventricular volumes and function	[11,21]
	Heart rate and heart rate variability	[22,34-37]
**Sleep and sleep deprivation**		
	Before race	[38,39]
	Nap	[39]
	Sleep structure before, during, and after	[38-40]
	Hallucination	[38]
	Vigilance	[38,41]
**Spatial cognition**		
	Posture	[20]
	Balance	[20,42]
**Shoes**		
	Pathologies	[43,44]
	Foot volume	[43,45]
**Running biomechanics**		
	Changes in kinetic parameters	[4,42,46-49]
**Myotendinous activity of the ankle joint**		
	Stiffness and fatigue	[50-53]
**Biological markers of inflammation**		
	Pro- and anti-inflammatory markers	[54-60]
	Sepsis markers, metabolism markers, and renal function markers	[61-66]
**Glycemia**		
	Control	[67]
	Role of food	[68]
**Psychology**		
	Motivation	[69,70]
**Profile and personality traits**		
	Skills for attentive presence to oneself, others, and the world	[71]
	Ability to project oneself in a future event	[71]
**Anthropometry**		
	Body fat	[72]
	Lean body mass	[25]
	BMI	[73]

### Race

The start date was given as November 11, 2021, at 2:30 PM. The race was divided into 6 identical loops of 26 km and 1000 m D+ and was run in semiautonomy; each runner had to be self-sufficient in water and food between each refreshment point. At the end of each loop, runners had access to a refreshment station identical to that of a classic race. After this refueling, runners moved to the scientific zone, and the stopwatch was paused for the duration of the scientific tests. Once the tests were completed, the runners started a new loop and the stopwatch was restarted.

Time barriers were set up based on calculations from similar races to replicate the constraints of a real race (Table 2).

The measurements were conducted from 36 to 3 hours before the start of the race for the prerace measurements, at the end of each lap, and continuously during the race for the per-race measurements, and then at the finish and from 24 hours to 10 days after the race for the postrace measurements.

If a participant dropped out of the race, the time and distance covered were recorded. The participant was then required to participate in the postrace testing.

Once the race was over, the time was recorded, and postrace measurements were taken within an hour. Measurements at +24 hours were also taken and followed up for a week after the race.

**Table 2:** Time barrier per lap.

Date	Last start time (time of day)	Lap	Last finish time (time of day)	Maximum time per lap (hours)	Minimum speed per lap (km/hour)	Science time (hours)
November 11, 2021	2:30 PM	1	7:30 PM	5	5.2	1
November 11 and 12, 2021	8:30 PM	2	2:30 AM	6	4.3	1
November 12, 2021	3:30 AM	3	10:30 AM	7	3.7	1
November 12, 2021	11:30 AM	4	7:30 PM	8	3.25	1
November 12 and 13, 2021	8:30 PM	5	5 AM	8.5	3	1
November 13, 2021	6 AM	6	3 PM	9	2.8	1

### Measures

#### Physiological Exploration

##### Anthropometry

Body mass was measured in kilograms using the BC545N (Tanita) scale. Body composition was assessed on the morning of the race and at the finish line using an mBCA 525 (Seca) impedance meter in the supine position to determine the proportion and distribution of fat, water, and muscle. It is a noninvasive technique validated against the gold standard [74].

##### Temperature

Body temperature analysis was performed by ingestion of an e-Celsius capsule (BodyCap). This is an ingested medical device that is noninvasive as it does not penetrate the skin or mucous membrane barrier and is connected to an external monitor that allows continuous measurement and recording of body temperature. The capsules are safe to ingest (17.7×8.9 mm, 1.7 g) and are eliminated through the natural route in 1 to 3 days in the stool. This device is valid, reproducible, and well-tolerated [6-9] and does not affect the athlete’s performance. The e-Celsius capsule also has a high T° accuracy of 0.2 °C.

On the morning of the race, at breakfast, the participants swallowed an e-Celsius capsule that allowed continuous measurement of core temperature. An e-Celsius skin patch was placed on a waistcoat that the runners had to wear throughout the race to record their body temperature. In case of expulsion, new capsules were activated and ingested whenever necessary. The chosen acquisition rate was 1 data point per minute.

Ambient temperature and humidity were measured using 2 Air Quality Transmitter AQT530 (Vaisala) weather stations installed on the course at the scientific base (0 km) and the halfway point (13 km).

##### Muscular Strength and Power

- Measurement of the maximum isometric strength of the knee extensors:
  Participants were seated on a quadriceps chair in a standardized position: arms crossed, hands on the shoulders, back in contact with the backrest, gaze horizontal, and knee angulation at 90° in the beginning. They had 2 alternative trials per leg (ie, 1 maximum repetition on the left, then on the right, then on the left, and again on the right). This measurement was repeated the day before, on the morning of the race, at the end of each loop, at the end of the race, and 24 hours after the race.
- Measurement of the maximum isometric strength of the hip abductors:
  Participants were placed in a side-lying position and were required to abduct against an inelastic strap set in a neutral position. A cushion was placed between the legs to position the hip in a neutral position. The first strap around the waist held the pelvis on the table to limit compensation from the trunk. A second strap was used as the dynamometer and was placed 5 cm above the external malleolus of the evaluated leg. After 2 submaximal tests, the participants were asked to perform at least three trials at their maximum strength, spaced by 1 minute of rest. This measurement was repeated the day before, on the morning of the race, and at the end of each loop.
- Measurement of the maximum isometric strength of the ankle plantar flexors:
  Participants were seated with their knees flexed at 90°. A rigid seatbelt strap was placed around the sole of the foot and secured to a step to provide resistance for the maximal test. The ankle position was maintained at 90° to ensure a stable ankle position with both the knee bent and straight. After 2 submaximal tests, the participants were asked to perform at least three trials at their maximum strength, spaced by 1 minute of rest. This measurement was repeated the day before, on the morning of the race, and at the end of each loop.
- Muscle power of the lower limbs:
  The day before the race, muscular power was evaluated using 3 squat jumps on a FD4000 force plate (Vald; 35 cm × 70 cm per plate). This measurement was repeated on the morning of the race, at the end of each lap, and at the end of the race. Each squat jump was separated by 30 seconds of rest. The instructions were as follows: “keep your hands on your hips and jump as high as possible during each repetition.”
- Grip force measurement:
  Maximal grip strength was evaluated using a grip dynamometer (Grip, K-Invent) the day before, on the morning of the race, at the end of each loop, and at the end. Participants sat in a chair with their arms in 90° elbow flexion. The instructions were to squeeze the dynamometer as hard as possible for 5 seconds. The rest time between trials was 30 seconds.

##### HR Measurement, Respiratory Rate, and HR Variability

The participants were equipped with a Hexoskin Pro Physiological Waistcoat (Carre Technologies Inc) to measure HR, HR variability, and respiratory rate during the night before the race, during the race, and during the 10 nights following the race.

##### Electrocardiography and Transthoracic Echocardiography

Electrocardiography and transthoracic echocardiography were performed on all participants the day before the race and at the end of the race or retirement. A subgroup of 30 runners, comprising 13 (43%) female athletes and 17 (57%) age-matched male athletes, was selected for an additional transthoracic echocardiography evaluation at the end of each lap. Blood pressure was measured after each echocardiographic examination with respect to 10 minutes of quiet rest, using an automated monitor (Omron) with an appropriate-sized arm cuff.

Echocardiographic assessment of cardiac volumes and function was conducted according to the current guidelines [75,76] using a commercially available echocardiographic system (Philips Epiq 7 equipped with an ×5-1 xMATRIX-array transducer). The examination was performed on site for each participant in the left lateral decubitus position using a standardized echocardiographic protocol. All echocardiographic measurements acquired during a brief apnea were stored digitally for offline data analysis, which will be performed by a single operator blinded to the study time point (TOMTEC-Arena TTA2, TOMTEC Imaging Systems GMBH). The left and right ventricular and atrial dimensions will be assessed using 2D parasternal and apical views. 3D ventricular volumes and ejection fractions will be obtained using TOMTEC 4D-analysis software. Ventricular and atrial deformations will be based on speckle-tracking analysis. Left ventricular relaxation will be analyzed using Doppler indices [76]. Left ventricular diastolic intraventricular pressure gradient, a marker of left ventricular suction, will be estimated noninvasively from echocardiographic color Doppler M-mode acquisitions made along the left ventricular base to apex axis in the 4-chamber apical view, as described previously [77,78].

##### Blood Biology

On the morning of the race, at the end of each loop, at the end of the race, and 24 hours after the race, venipuncture was performed on participants in a sitting position. Blood samples (2 mL) were collected from the forearm in heparin and citrate tubes. The samples were centrifuged and aliquoted for further analysis. Coagulant-free serum, serum EDTA, and heparinized vacutainers allowed us to obtain serum and plasma for the following further analyses:

- Plasma levels of interleukin (IL)-1, IL-6, tumor necrosis factor (TNF)-α, protein S100, neuron-specific enolase, C-reactive protein (proinflammatory markers), and IL-4, IL-10, and IL-13 (anti-inflammatory markers)
- The parameters studied related to sepsis will be granzyme B, heat shock protein 70, IL-1α, IL-8, macrophage inflammatory protein 1 α, macrophage inflammatory protein 1 β, and matrix metalloproteinase-8
- The studied parameters related to metabolism will be ghrelin, gastric inhibitory polypeptide, glucagon-like peptide-1, glucagon, insulin, insulin leptin plasminogen activator inhibitor-1 (total), resistin, visfatin, C-peptide, cortisol, pancreatic polypeptide, insulin, and peptide YY
- The parameters studied related to inflammation will be soluble CD30, soluble epidermal growth factor receptor, soluble glycoprotein 130, soluble IL (sIL)-1 receptor type I, sIL-1 receptor type II, sIL-2 receptor type α, sIL-4 receptor, sIL-6 receptor, advanced glycosylation end product-specific receptor, soluble TNF receptor I, soluble TNF receptor II, soluble vascular endothelial growth factor (sVEGF) receptor 1, sVEGF receptor 2, and sVEGF receptor 3
- The parameters studied in relation to renal function will be blood count and blood ionogram with calcium, phosphorus, magnesium, urea, creatinine, neutrophil gelatinase-associated lipocalin, kidney injury molecule 1, plasma, and urine lipocalin

Venous blood gas analysis was immediately performed using the Stat Profile Prime (Nova Biomedical) medical device, allowing hemoglobin measurement and hematocrit calculation.

##### Glycemia

The day before the race, the investigators placed a continuous interstitial glucose sensor (FreeStyle Libre Pro, Abbott) on the back of participants’ arms. This sensor was used in masked mode; hence, the runners did not have live access to their blood glucose values, so as not to interfere with their usual running strategies. The sensor is self-calibrating and does not need to be manipulated once fitted. Blood glucose levels were estimated from interstitial glucose levels measured at 15-minute intervals from the time the sensor was fitted until it was removed 9 days after the race.

In addition, plasma glucose levels were analyzed from venous blood samples collected on the morning before the race, at the end of each loop, at the end of the race, and 24 hours after the race.

##### Sleep Exploration

A sleep questionnaire, adapted from the existing Spiegel, Epworth, and Vis-Morgen questionnaires, was administered to all participants before, during (if a nap was required), and after the race.

On the night before the race and the 7 nights after the race, the participants were equipped with the Hexoskin Pro Waistcoat, measuring sleep indirectly by actimetry.

Sleep was also recorded by electroencephalogram measurements using a Somfit (Compumedics Limited) for naps during the race and in the 7 nights following the race for a subgroup.

On the day before the race, the day of the start of the race, at the end of each loop, and at the end of the race or retirement, participants were asked to complete the Karolinska Subjective Sleepiness Scale.

#### Biomechanical Exploration

##### Elastic and Architectural Properties of the Gastrocnemius-Achilles Tendon Complex

Ultrasound examination was performed using a linear array transducer (EPIQ Elite with eL18-4 transducer ElastQ Imaging shear wave elastography, Philips Medical Systems). All participants were examined in the prone position, with the knee in the extended position and the ankle fixed in a neutral position. Both legs were assessed. The cross-sectional area of the Achilles tendon (in mm^2^) was measured at the level between the malleoli [79]. Longitudinal panoramic sonographic images of the medial gastrocnemius muscle were obtained. Pennation angle and fascicle length were measured at the middle and distal parts of the muscle [80]. The elastic properties of the medial gastrocnemius muscle were measured in the longitudinal view at 30% of the muscle length using Young modulus values (in kPa) determined by shear wave ultrasound elastography [81]. The region of interest circle was placed in the muscle belly, and median elasticity, maximum elasticity, and average elasticity were collected. Measurements were performed before the race and repeated at the end of the second lap, the fourth lap, at the end or at retirement, and 6 hours after the race.

##### Running Kinetics and Kinematics

The data were collected on the day before the Trail Scientifique Clécy (10 minutes of warm-up before data collection for a few seconds for the kinematics); during the race; at the end of each loop; and 30 m before the end of a lap on a flat, paved, and covered portion.

The kinematics of the race were evaluated using a high-definition video camera and an Optojump system (Microgate) at the end of each loop. The characterization of foot strike patterns (rear foot, midfoot, and forefoot) using a video camera is a valid and accurate method of assessment [82-84]. The foot strike angle at the initial contact was also measured using a high-speed, high-definition camera at 240 frames per second. Step rate, step length, and ground and fly contact time were measured using an Optojump system comprising fixed sensors of 15 m × 1.5 m, which were positioned in an 18 m × 3 m tent on a level section. This instrument has been validated against an instrumented treadmill [85].

RunScribe sensors were used continuously throughout the run to measure the kinematic parameters of the stride. The variables of interest were the foot strike pattern, power, flight time, flight ratio, step rate, and ground contact time, which were previously found to be valid and reliable [86].

The loops were broken down into sections (ascent, descent, and flat) to isolate the variables and analyze the intermediate race times.

##### Shoes

The day before the race, information was collected on the trail shoes used by the participants (brand, model, size, weight, sole thickness and drop, torsional and longitudinal flexibility, motion control technologies, and the Minimalist Index [87]). A questionnaire was also administered to define their needs, expectations, and preferences regarding the footwear used.

##### Foot Measurement

Both feet were scanned using a photogrammetric 3D scanner (FeetBox3D, Corpus-e). A 3D model of the feet was reconstructed to observe the structural changes induced by the race (swelling).

This measurement was taken before the race and repeated at the end of each lap and at the end of the race or retirement.

#### Cognitive, Psychological, and Sociological Exploration

##### Vestibular System: Testing Sensory Organization and Measuring Spatial Orientation

We assessed verticality perception to evaluate the visuovestibular sensory preference with subjective vertical visual, dynamic subjective vertical visual, and the Rode and Frame test 4 hours before the race, at loops 1 and 3, at the end loop 6, and 24 hours after the race.

We measured the spatial strategy according to the egocentric (striatal network) versus allocentric (hippocampal network) response through the reverse T maze previously performed in rodents [88] and in healthy participants [49] before the race, at the end of loop 6, and at 24 hours after the race.

All tests were performed using a virtual reality headset setup (VRMaze [89]).

##### Postural Control

Postural control was measured 2 times for 50 seconds (open and closed eye conditions) on K-Force Plates (K-Invent).

This measurement was repeated 24 hours and 3 hours before the race, at loops 1 and 3, at the end of loop 6, and 24 hours after the race. Anteroposterior and lateral sway and stability scores were analyzed.

##### Cognitive Tests

On the day before the race, the morning of the race, at the end of each loop, and then at the end or at retirement, the runners were asked to assess a simple 5-minute serial response time test [90]. The number of mistakes termed *errors of omission* (eg, lapses of attention, historically defined as response time ≥500 milliseconds) plus *errors of commission* (eg, responses without a stimulus, false starts, or response time <100 milliseconds) are the primary outcome measures. The mean response times were also calculated. A measure of perceived sleepiness using the Karolinska Sleepiness Scale completed the objective assessment.

##### Psychomotivational Test

On the morning of the race, at the end of each loop, at the finish line, and 24 hours after the race, participants completed a motivational test.

The methodology comprised asking participants to perform 2 tests on a computer, each lasting approximately 3 minutes. A long version of these tests was described by Schmidt et al [91]. One was for physical effort, and the other was for mental effort. The short version was implemented during the Reunion ultratrail (Grand Raid).

In each test, participants were asked to try to win as much money as possible. The money was not real, as in a video game; however, the amount won is used to rank participants. Each trial had a coin or note (10c or 0.11c, €1 or US $1.06, or €10 or US $10.58) that one can win if they do their best. The maximum was specific to each participant and was measured during the prerace visit, which allowed instructions to be given.

Each test comprised 9 trials, 3 per incentive level, where the participant must either squeeze the handle as hard as possible or solve as many numerical comparisons as possible in a limited time. At the end of each trial, the participant was told his or her performance and the amount of money earned, calculated as the fraction of the incentive corresponding to the fraction of the maximum effort achieved. The maximum effort corresponds to the maximum muscular contraction produced during the calibration visit and the minimum time taken to complete 10 numerical comparisons.

##### Sociological Questionnaire

Before the race, the participants had to fill in a sociological questionnaire on their ability to be attentive to themselves, others, and the world using various indicators.

#### Environmental Exploration

To measure the air quality, we installed 2 sensors (Air Quality Transmitter AQT530, Vaisala) along the course of the trail of Clécy at a height of 1.70 m from the ground. The sensors were placed at 2 locations along the course. The first sensor was located at the center of the Pleine Nature Lionel Terray at the start of the race. This point was also the passage of each 26 km loop and the arrival of the race. This was the point of the course with the lowest altitude (50 m). The second sensor was located at the aid station at the 14th kilometer of the course, which was the point of the course with the highest altitude (254 m). The frequency of the measurement of the sensors was 1 measurement every 10 minutes.

These sensors measured nitrogen dioxide, nitrogen monoxide, carbon monoxide, ozone, and fine particles (particulate matter [PM]) with diameters <1 µm (PM1), <2.5 µm (PM2.5), and <10 µm (PM10). Environmental parameters such as temperature, humidity, and atmospheric pressure were also recorded.

#### Other Measures

Crosscall Core T4 tablets (France) with Quicktape Survey software (Canada) were used to merge the questionnaires of each scientific team.

#### Order of Tasks

To reduce the impact of one measure on another for the prerace measurements, the order of passage of the tasks was imposed. Therefore, no randomization was performed.

**Figure 1:**
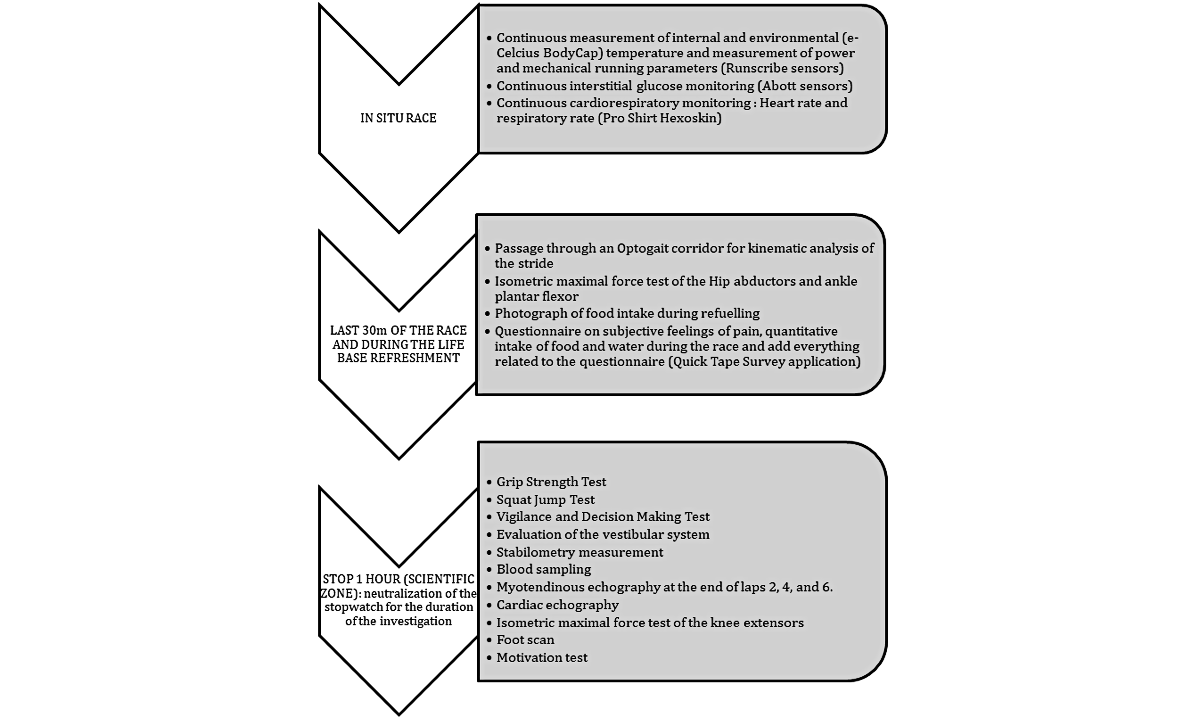
Organization of scientific measurements.

Figure 1 shows the sequence of the scientific part.

### Statistical Analysis

Data related to the primary and secondary objectives will be evaluated at the end of the study. For each studied parameter, the normality of the distribution will be examined using the Shapiro-Wilk test. Continuous quantitative variables will be expressed as mean (SD), discontinuous variables as median and IQR, and qualitative variables as percentages. The comparison of the values of the primary end point between the baseline and the end of the study will be performed using statistical tests for the paired series. In addition, to take into account repeated measures over time, we will analyze the primary end point as a function of time using appropriate mixed models for repeated data. Using the same statistical tests as for the primary end point, we will analyze the secondary end points. The threshold for statistical significance will be defined as *P*˂.05. Depending on the scientific team, we will use MedCalc Statistical Software (version 13.2.0) and JASP (version 0.16.1.0) or RStudio (version 1.2) to perform the statistical analyses.

### Ethics Approval

The launch of the study was authorized on October 26, 2021, under trial number 21-0166 after a favorable opinion from the Comité de Protection des Personnes Ouest III (21.09.61/SIRIPH 2G 21.01586.000009).

## Results

### Overview

Of the 60 runners selected for the scientific trail, 56 (93%) showed up, and 55 (92%) were selected for the study, including 43 (72%) men (mean age 45.6, SD 14.6 years; mean height 1.76 m, SD 0.1 m; mean weight 70.3 kg, SD 7.8 kg; mean BMI 22.7, SD 2.0; and mean body fat 9.7%, SD 5.4%) and 12 (20%) women (mean age 43.8, SD 9.7 years; mean weight 53.5, SD 5.5 kg; mean BMI 19.7, SD 1.1; mean body fat 17.7%, SD 4.8%).

A woman was excluded from the study because of her participation in a 160 km ultramarathon 1 week before the protocol.

There were 14 participants who abandoned the study for the following reasons: perceived hypothermia (n=2, 14%), generalized exhaustion (n=5, 36%), gastric problems (n=4, 29%), and musculoskeletal pain (n=3, 21%).

We performed intermediate times over the entire race. Table 3 lists the values. Race time corresponds to the time taken to complete the 6 loops. Stop time corresponds to the time spent at the base of life (refueling and paramedical care). Science time refers to the time spent on various scientific measurements.

In a classic trail-running race, running and stopping times are part of the final timing. Here, this value is represented by the total time.

For finishers, the average time for science over the whole race was 320 (SD 56) minutes (ie, an average of 64, SD 13 minutes per lap; ie, 17.4%, SD 2.1% of the protocol time). As an indication, the time spent at the base of life for refreshments, a nap, foot care, and physiotherapy represented 8.6% (SD3.7%) of the protocol time.

For did not finish, the science time corresponded to 18.2% (SD 3.9%) of the protocol time, and 8.6% and SD 3.5%) of the protocol time was devoted to stops at the base of life.

The average time per loop is presented in Table 4 for all the runners, finishers, and did not finish.

**Table 3:** Timing and split times.

Participants	Race time (hours)	Break time (hours)	Science time (hours)	Total time (race time+break time; hours)	Protocol time (total time+science time; hours)
Finishers, mean (SD)	23.3522 (3.5197)	2.1611 (1.3147)	5.3481 (0.9392)	1.4967 (4.5661)	30.8447 (5.2439)
First man	17.1839	0.6367	3.8969	17.8206	21.7175
First woman	19.7478	1.0992	4.8147	20.8469	25.6617

**Table 4:** Average time per lap (26 km and 1000 m D+) for the group, finishers, and nonfinishers (N=55).

Participants	Lap 1	Lap 2	Lap 3	Lap 4	Lap 5	Lap 6
Runners at each lap, n (%)	55 (100)	55 (100)	53 (96)	50 (91)	44 (80)	41 (75)
Time (all runners n=54), mean (SD)	3.0119 (0.3344)	3.5217 (0.4306)	3.9736 (0.6708)	4.2397 (0.6608)	4.4114 (0.9072)	4.6444 (0.9625)
Time for finishers (n=41), mean (SD)	2.9633 (0.35)	3.4528 (0.4197)	3.8631 (0.6214)	4.1261 (0.5825)	4.3144 (0.8425)	4.6444 (0.9625)
Time for did not finish, mean (SD)	3.1536 (0.2419)	3.7236 (0.4117)	4.3511 (0.7231)	4.7578 (0.78)	5.7367 (0.8175)	N/A^a^

^a^N/A: not applicable (there are no more runners in this category on lap 6 as they did not finish the race).

### Undesirable Effects

No runner had to interrupt the race because of an undesirable effect of the protocol.

We noted a few lipothymia cases, which were not serious, during certain scientific tests, particularly those requiring prolonged standing.

We did not note any muscle damage related to the tests.

The venipunctures generated ecchymosis because of the difficulty of puncturing but also extravasation secondary to the rapid resumption of the race (strong and lasting compression not being possible).

Anecdotally, 8 punctures, 6 of which were during the race, were a major constraint.

## Discussion

### Principal Findings

The Trail Scientifique de Clécy allowed, for the first time, an integrative and multidisciplinary approach to better understand the performance of ultramarathons. The hypotheses based on the observations of the studies conducted in trail running go in the direction of degradation of many functions in the first one-third of the race before reaching a plateau in the last one-third of the race, suggesting that the differences in certain parameters of tiredness are no longer significant between the arrival of 100 miles or 200 miles in a mountain [3,4,20,41,42,48,92-94].

Thus, our initial hypothesis was that there is a significant degradation of all the studied functions under the effect of race-induced fatigue from the first one-third of the race before reaching a plateau on the third one-third. We assume that a difference or difference in the kinetics of the studied functions can lead to failure and abandonment.

Owing to its duration, a mountain ultramarathon induces night and day phases, which can induce a circadian effect on the fluctuation of physiological and cognitive functions. Our team is interested in verifying whether there is a circadian effect on the kinetics of the functions studied during the Trail Scientifique de Clécy, according to the running time and the time of day.

Our study population is representative of the real population of a classic ultratrail race. Our average age (45, SD 13.6 years) is in line with the recent observations, who reported that the average age of the different races of the Ultra Trail du Mont Blanc (Courmayeur Champex Chamonix and Orcières Champex Chamonix) from 2014 to 2018 for the 40 to 49 age group is the most represented [95]. Concerning the distribution of men and women, 21.8% of women against 9.8% of women are on the starting line for the Ultra Trail du Mont Blanc 2021 [95].

The downtime imposed to meet the requirements of the scientific protocol did not exceed 20% of the total time. These data allow us to justify that our scientific race model is closer to reality because of the reduced duration of the stopping time for scientific tasks compared with the duration of the race and voluntary stops.

### Conclusions

The originality of this project is that it has allowed an integrative approach to the different parameters determining performance in ultraendurance. Our protocol allowed us to standardize the measurement points with a fixed and identical loop for each lap to collect fixed or continuous measurement points over the entire course, which is not the case in rare studies conducted in racing [5-26]. The loops can be compared with each other and will allow for splitting into sections to analyze each section 6 times in a row.

The first results for each scientific task are expected in June 2022.

## Appendix

Multimedia Appendix 1 Peer review report by the Agence nationale de la recherche (ANR, or French National Research).

Multimedia Appendix 2 Statement of expertise HAIS N°1.

Multimedia Appendix 3 Statement of expertise HAIS N°2.

Multimedia Appendix 4 Statement of expertise HAIS N°3.

## Abbreviations

HR: heart rate
IL: interleukin
PM: particulate matter
sIL: soluble interleukin
sVEGF: soluble vascular endothelial growth factor
TNF: tumor necrosis factor
