# Supplementary material for: Study of the Kinetics of the Determinants of Performance During a Mountain Ultramarathon: Multidisciplinary Protocol of the First Trail Scientifique de Clécy 2021
Source: JMIR Res Protoc. 2022 Jun 15;11(6):e38027. doi: 10.2196/38027 (PMC9244647; doi:10.2196/38027)
Supplement: Multimedia Appendix 1 [file resprot_v11i6e38027_app1.pdf]

**CP D 20-11-32**

RÉGION
NORMANDIE

Le Président

# RÉGION NORMANDIE

## Commission Permanente
## Réunion du 16 novembre 2020

**14h00, à Caen en visioconférence**

**Sous la présidence de Monsieur MORIN**

**DELIBERATION**

| | |
|---|---|
| **Objectif stratégique** | **Pour une économie normande dynamique, attractive et innovante** |
| **Mission** | **Accompagner la recherche et l'innovation, levier de développement économique** |
| **Programme** | **P270 - Soutenir la recherche** |
| ***Titre*** | **SOUTIEN AUX PROJETS « TREMPLINS » - RIN RECHERCHE 2020** |

Présents :
Céline BRULIN, Anne-Marie COUSIN, Hubert DEJEAN DE LA BATIE, Bertrand DENIAUD, Clotilde EUDIER, Jean-Baptiste GASTINNE, Sophie GAUGAIN, Patrick GOMONT, Françoise GUEGOT, Franck GUEGUENIAT, Marie-Françoise GUGUIN, Chantal HENRY, Timothée HOUSSIN, Marie-Françoise KURDZIEL, Guy LEFRAND, David MARGUERITTE, Florence MAZIER, Hervé MORIN, Marie-Agnès POUSSIER-WINSBACK, Claire ROUSSEAU, Claude TALEB, Rodolphe THOMAS.

Excusés et pouvoirs :
Laurent BEAUVAIS, Hélène BURGAT, Claudie LAUNOY, Nicolas MAYER-ROSSIGNOL, Francine LAVANRY (pouvoir à Timothée HOUSSIN), Hervé MAUREY (pouvoir à Hervé MORIN), Joachim MOYSE (pouvoir à Céline BRULIN), Jean-Jacques NOEL (pouvoir à Marie-Françoise KURDZIEL), Guillaume PENNELLE (pouvoir à Chantal HENRY), Alexandra PIEL (pouvoir à Timothée HOUSSIN), Gaëlle PIOLINE (pouvoir à Franck GUEGUENIAT), François-Xavier PRIOLLAUD (pouvoir à Sophie GAUGAIN).

Vu les articles L 4211-1, L 4221-1 et L 4221-5 du Code Général des Collectivités Territoriales,

Vu la décision du 5 décembre 2014 de la Commission européenne relative à l’approbation du programme opérationnel FEDER/FSE de la Basse-Normandie,

Vu la décision du 12 décembre 2014 de la Commission européenne relative à l’approbation du programme opérationnel FEDER/FSE de Haute-Normandie,

Vu la décision n° C(2019)6769 de la Commission européenne en date du 20 septembre 2019 adoptant la modification du Programme Opérationnel FEDER-FSE Haute-Normandie 2014-2020 n° CCI 2014FR16M0OP005, modifié par l'Etat membre le 23 mai 2019 et entré en vigueur le 29 mai 2019,

Vu la décision n° C(2019)6770 de la Commission européenne en date du 20 septembre 2019 adoptant la modification du Programme Opérationnel FEDER-FSE Basse-Normandie 2014-2020 n° CCI 2014FR16M2OP001, modifié par l'Etat membre le 23 mai 2019 et entré en vigueur le 29 mai 2019,

Vu la délibération n° AP D 16-12-1 du 15 décembre 2016 portant approbation des Schémas Régionaux de Développement Economique des Entreprises pour l'Innovation et l'Internationalisation (SRDEEII) et de l'Enseignement Supérieur de la Recherche et de l'Innovation (SRESRI),

Vu la délibération n° AP D 17-12-9 du Conseil Régional en date du 18 décembre 2017 modifiant le règlement budgétaire et financier de la Région, adopté par délibération n° AP D 16-03-19 du 24 mars 2016,

Vu la délibération n° AP D 19-03-16 du Conseil Régional en date du 18 mars 2019 modifiant la délégation du Conseil Régional à la Commission Permanente, adoptée par délibération n° AP D 17-11-14 du 20 novembre 2017,

Vu la délibération n° CP D 19-07-1 de la Commission Permanente en date du 4 juillet 2019 modifiant le règlement des subventions régionales, adopté par délibération n° AP D 16-03-20 du 24 mars 2016, et modifiant les modèles de conventions génériques pour les aides régionales soumises à ce règlement, adoptés par délibération n° AP D 18-10-20 en date du 15 octobre 2018,

Vu la délibération n° CP D 19-11-30 du Conseil Régional du 18 novembre 2019 modifiant la délibération n° AP D 17-02-3 du Conseil Régional en date du 6 février 2017 approuvant le dispositif RIN Recherche,

Vu la délibération n° AP D 19-12-6 du Conseil Régional en date du 16 décembre 2019 adoptant le Budget Primitif 2020 du Budget principal, le Conseil Economique, Social et Environnemental Régional consulté,

Vu la délibération n° AP D 20-06-9 du Conseil Régional en date du 22 juin 2020 adoptant le Budget Supplémentaire 2020 du Budget Principal, le Conseil Economique, Social et Environnemental Régional consulté,

Vu la délibération n° AP D 20-10-13 du Conseil régional en date du 12 octobre 2020 adoptant la décision modificative n°1 du Budget Principal 2020, le Conseil Economique, Social et Environnemental Régional consulté,

Vu la délibération de la Commission permanente en date du 14 septembre 2020, modifiant la délibération n° CP D 20-07-23 en date du 06 juillet 2020 et approuvant la convention-type RIN Recherche,

**Considérant :**

Qu'en déclinaison du Schéma Régional de l'Economie, des Entreprises, de l'internationalisation et de l'innovation (SRDE*E*II) et du Schéma Régional de l'Enseignement Supérieur, de la Recherche et de l'Innovation (SRESRI) adoptés à l'Assemblée plénière du Conseil Régional du 15 décembre 2016, la Région souhaite conforter les axes de recherche d'excellence et en émergence sur le territoire normand, en finançant des projets de recherche afin :

- d'accroitre la visibilité et l'attractivité du potentiel de la recherche du territoire normand, tout en irrigant le tissu économique de la région,
- promouvoir des projets d'excellence scientifique, originaux et d'intérêt pour la Normandie,

- permettre aux équipes de recherche de se structurer pour atteindre une reconnaissance et une visibilité européenne et internationale qui renforcera l'attractivité de la Normandie.

Que la Région, dans le cadre du dispositif RIN Recherche tel qu'adopté le 18 novembre 2019, entend soutenir l'excellence et l'attractivité des laboratoires de recherche normands en cohérence avec la stratégie des établissements, des organismes et de la COMUE Normandie Université à travers des projets tremplins qui favorisent le développement de nouvelles thématiques de recherche,
Le dépôt de demandes de soutien de projets de recherche, détaillés en annexe 3 et répondant aux objectifs du dispositif RIN Recherche,

Après en avoir délibéré,

**Décide**, à l'unanimité des voix,

- de procéder aux affectations pour un montant total de 5 270 295,00 € présentées l'annexe 1 ;

- d'attribuer un montant total de 5 270 295,00 € aux bénéficiaires pour leurs projets de recherche Tremplins, selon l'annexe 2 ;

- de déroger à la convention type FEDER et à la convention-type RIN Recherche en autorisant une date limite de transmission des pièces justificatives 4 mois après la date de fin de l'opération ;

- d'autoriser le Président à signer avec les bénéficiaires listés en annexe 2 :
    - les conventions RIN Recherche selon le modèle adopté par la Commission Permanente en date du 14 septembre 2020 ;
    - les conventions FEDER selon le modèle adopté par délibération n° CP D 18-06-5 en date du 4 juin 2018 ;
    - tous les actes utiles à la mise en œuvre de ces décisions.

*Hervé MORIN*

Acte rendu exécutoire le 24 novembre 2020
après réception Préfecture le 24 novembre 2020
Référence technique : 076-200053403-20201116-103127-DE-1-1
et affichage ou notification le 24 novembre 2020

*Dans les deux mois à compter de sa publication ou de sa notification, cette délibération est susceptible de recours devant le tribunal administratif de Caen. Le tribunal administratif peut être saisi par l'application Télérecours citoyens, accessible par le site* www.telerecours.fr.

Annexe 1

**REC - Programme P270 - Soutenir la recherche**

**Opération P270O002 Projets de recherche**

| Code Ttranche | Description Tranche | Code AP/EPCP | Libellé AP/EPCP | Mt Pluri affect. | N° Mvt Tranche | Statut Mvt Tranche | Montant Mvt Tranche |
|---|---|---|---|---|---|---|---|
| 20E05189T01 | FEDER - CNRS - SO SIRIUS - Tranche 01 | P270E07-EBN | AE-2015-D-936-FEDER-BN-Souteni | 0,00 | Affectation initiale | Prévisionnel | 144 000,00 |
| 20E05904T02 | FEDER - LABEO - AVEq-ACTIVE - Tranche 02 | P270E07-EBN | AE-2015-D-936-FEDER-BN-Souteni | 0,00 | Affectation initiale | Prévisionnel | 120 500,00 |
| 20E05909T02 | FEDER - ANSES - AVEq-ACTIVE - Tranche 02 | P270E07-EBN | AE-2015-D-936-FEDER-BN-Souteni | 0,00 | Affectation initiale | Prévisionnel | 76 000,00 |
| 20E06082T01 | FEDER - UNICAEN - EYE TRACKING - Tranche 01 | P270E07-EBN | AE-2015-D-936-FEDER-BN-Souteni | 0,00 | Affectation initiale | Prévisionnel | 77 500,00 |
| 20E06083T01 | FEDER - UNICAEN - SCHISM - Tranche 01 | P270E07-EBN | AE-2015-D-936-FEDER-BN-Souteni | 0,00 | Affectation initiale | Prévisionnel | 114 000,00 |
| 20E06084T02 | FEDER - UNICAEN - ETHNOTEVE - Tranche 02 | P270E07-EBN | AE-2015-D-936-FEDER-BN-Souteni | 0,00 | Affectation initiale | Prévisionnel | 5 000,00 |
| 20E06086T02 | FEDER - UNICAEN - DYNET - Tranche 02 | P270E07-EBN | AE-2015-D-936-FEDER-BN-Souteni | 0,00 | Affectation initiale | Prévisionnel | 110 000,00 |
| 20E06087T01 | FEDER - UNICAEN - HAIS - Tranche 01 | P270E07-EBN | AE-2015-D-936-FEDER-BN-Souteni | 0,00 | Affectation initiale | Prévisionnel | 559 951,00 |
| 20E06088T05 | FEDER - UNICAEN - PredicAlert - Tranche 05 | P270E07-EBN | AE-2015-D-936-FEDER-BN-Souteni | 0,00 | Affectation initiale | Prévisionnel | 280 700,00 |
| 20E06089T02 | FEDER - ENSICAEN - PredicAlert - Tranche 02 | P270E07-EBN | AE-2015-D-936-FEDER-BN-Souteni | 0,00 | Affectation initiale | Prévisionnel | 59 500,00 |
| 20E06128T02 | FEDER - ENVA - OA - ACTIVE - Tranche 02 | P270E07-EBN | AE-2015-D-936-FEDER-BN-Souteni | 0,00 | Affectation initiale | Prévisionnel | 94 850,00 |
| 20E06163T01 | FEDER - UNICAEN - OA-ACTIVE - Tranche 01 | P270E07-EBN | AE-2015-D-936-FEDER-BN-Souteni | 0,00 | Affectation initiale | Prévisionnel | 202 000,00 |
| 20E06170T01 | FEDER - UNICAEN - RN-LEGO - Tranche 01 | P270E07-EBN | AE-2015-D-936-FEDER-BN-Souteni | 0,00 | Affectation initiale | Prévisionnel | 107 500,00 |
| | | | | | | ***Sous-Total P270E07-EBN :*** | **1 951 501,00** |

| Code Ttranche | Description Tranche | Code AP/EPCP | Libellé AP/EPCP | Mt Pluri affect. | N° Mvt Tranche | Statut Mvt Tranche | Montant Mvt Tranche |
|---|---|---|---|---|---|---|---|
| 20E05904T01 | FEDER - LABEO - AVEq-ACTIVE - Tranche 01 | P270E16-EBN | AP-2015-D-906-FEDER-BN-Souteni | 0,00 | Affectation initiale | Prévisionnel | 40 000,00 |
| 20E05909T01 | FEDER - ANSES - AVEq-ACTIVE - Tranche 01 | P270E16-EBN | AP-2015-D-906-FEDER-BN-Souteni | 0,00 | Affectation initiale | Prévisionnel | 26 500,00 |

| | | | | | | | |
|---|---|---|---|---|---|---|---|
| 20E06082T02 | FEDER - UNICAEN - EYE TRACKING - Tranche 02 | P270E16-EB | AP-2015-D-906-FEDER-BN-Souteni | 0,00 | Affectation initiale | Prévisionnel | 3 000,00 |
| 20E06083T02 | FEDER - UNICAEN - SCHISM - Tranche 02 | P270E16-EB | AP-2015-D-906-FEDER-BN-Souteni | 0,00 | Affectation initiale | Prévisionnel | 4 000,00 |
| 20E06084T01 | FEDER - UNICAEN - ETHNOTEVE - Tranche 01 | P270E16-EB | AP-2015-D-906-FEDER-BN-Souteni | 0,00 | Affectation initiale | Prévisionnel | 250 000,00 |
| 20E06086T01 | FEDER - UNICAEN - DYNET - Tranche 01 | P270E16-EB | AP-2015-D-906-FEDER-BN-Souteni | 0,00 | Affectation initiale | Prévisionnel | 4 000,00 |
| 20E06087T02 | FEDER - UNICAEN - HAIS - Tranche 02 | P270E16-EB | AP-2015-D-906-FEDER-BN-Souteni | 0,00 | Affectation initiale | Prévisionnel | 92 700,00 |
| 20E06088T03 | FEDER - UNICAEN - PredicAlert - Tranche 03 | P270E16-EB | AP-2015-D-906-FEDER-BN-Souteni | 0,00 | Affectation initiale | Prévisionnel | 47 900,00 |
| 20E06089T01 | FEDER - ENSICAEN - PredicAlert - Tranche 01 | P270E16-EB | AP-2015-D-906-FEDER-BN-Souteni | 0,00 | Affectation initiale | Prévisionnel | 2 000,00 |
| 20E06128T01 | FEDER - ENVA - OA - ACTIVE - Tranche 01 | P270E16-EB | AP-2015-D-906-FEDER-BN-Souteni | 0,00 | Affectation initiale | Prévisionnel | 2 600,00 |
| 20E06170T02 | FEDER - UNICAEN - RN-LEGO - Tranche 02 | P270E16-EB | AP-2015-D-906-FEDER-BN-Souteni | 0,00 | Affectation initiale | Prévisionnel | 15 000,00 |
| | | | | | | ***Sous-Total P270E16-EBN :*** | **487 700,00** |
| **Code Ttranche** | **Description Tranche** | **Code AP/EPCP** | **Libellé AP/EPCP** | **Mt Pluri affect.** | **N° Mvt Tranche** | **Statut Mvt Tranche** | **Montant Mvt Tranche** |
| 20E06177T02 | REGION - PRAXENS - RN-LEGO - Tranche 02 | P270E30 | AE-2020-D-939-Soutenir la rech | 0,00 | Affectation initiale | Prévisionnel | 15 000,00 |
| | | | | | | ***Sous-Total P270E30 :*** | **15 000,00** |
| **Code Ttranche** | **Description Tranche** | **Code AP/EPCP** | **Libellé AP/EPCP** | **Mt Pluri affect.** | **N° Mvt Tranche** | **Statut Mvt Tranche** | **Montant Mvt Tranche** |
| 20E06177T01 | REGION - PRAXENS - RN-LEGO - Tranche 01 | P270E31 | AP-2020-D-909-Soutenir la rech | 0,00 | Affectation initiale | Prévisionnel | 10 000,00 |
| | | | | | | ***Sous-Total P270E31 :*** | **10 000,00** |
| **Code Ttranche** | **Description Tranche** | **Code AP/EPCP** | **Libellé AP/EPCP** | **Mt Pluri affect.** | **N° Mvt Tranche** | **Statut Mvt Tranche** | **Montant Mvt Tranche** |
| 20E02190T01 | FEDER - CNRS - HAITY - Tranche 01 | P270E34-EH | AE-2015-D-936-FEDER-HN-Souteni | 0,00 | Affectation initiale | Prévisionnel | 213 000,00 |
| 20E05832T01 | FEDER - INSA - EFLUX - Tranche 01 | P270E34-EH | AE-2015-D-936-FEDER-HN-Souteni | 0,00 | Affectation initiale | Prévisionnel | 346 260,00 |
| 20E05838T01 | FEDER - URN - SCHISM - Tranche 01 | P270E34-EH | AE-2015-D-936-FEDER-HN-Souteni | 0,00 | Affectation initiale | Prévisionnel | 113 000,00 |
| 20E05935T02 | FEDER - URN - EYE TRACKING - Tranche 02 | P270E34-EH | AE-2015-D-936-FEDER-HN-Souteni | 0,00 | Affectation initiale | Prévisionnel | 82 500,00 |

| | | | | | | | |
|---|---|---|---|---|---|---|---|
| 20E05972T02 | FEDER - ESIGELEC - ETHNOTEVE - Tranche 02 | P270E34-EHN | AE-2015-D-936-FEDER-HN-Souteni | 0,00 | Affectation initiale | Prévisionnel | 72 500,00 |
| 20E06110T02 | FEDER - ULHN - DYNET - Tranche 02 | P270E34-EHN | AE-2015-D-936-FEDER-HN-Souteni | 0,00 | Affectation initiale | Prévisionnel | 110 000,00 |
| 20E06174T02 | FEDER - URN - RN-LEGO - Tranche 02 | P270E34-EHN | AE-2015-D-936-FEDER-HN-Souteni | 0,00 | Affectation initiale | Prévisionnel | 312 000,00 |
| | | | | | | ***Sous-Total P270E34-EHN :*** | **1 249 260,00** |

| Code Ttranche | Description Tranche | Code AP/EPCP | Libellé AP/EPCP | Mt Pluri affect. | N° Mvt Tranche | Statut Mvt Tranche | Montant Mvt Tranche |
|---|---|---|---|---|---|---|---|
| 20E02190T02 | FEDER - CNRS - HAITY - Tranche 02 | P270E35-EHN | AP-2015-D-906-FEDER-HN-Souteni | 0,00 | Affectation initiale | Prévisionnel | 282 000,00 |
| 20E05832T02 | FEDER - INSA - EFLUX - Tranche 02 | P270E35-EHN | AP-2015-D-906-FEDER-HN-Souteni | 0,00 | Affectation initiale | Prévisionnel | 90 000,00 |
| 20E05838T02 | FEDER - URN - SCHISM - Tranche 02 | P270E35-EHN | AP-2015-D-906-FEDER-HN-Souteni | 0,00 | Affectation initiale | Prévisionnel | 3 000,00 |
| 20E05935T03 | FEDER - URN - EYE TRACKING - Tranche 03 | P270E35-EHN | AP-2015-D-906-FEDER-HN-Souteni | 0,00 | Affectation initiale | Prévisionnel | 26 000,00 |
| 20E05972T01 | FEDER - ESIGELEC - ETHNOTEVE - Tranche 01 | P270E35-EHN | AP-2015-D-906-FEDER-HN-Souteni | 0,00 | Affectation initiale | Prévisionnel | 70 000,00 |
| 20E06110T01 | FEDER - ULHN - DYNET - Tranche 01 | P270E35-EHN | AP-2015-D-906-FEDER-HN-Souteni | 0,00 | Affectation initiale | Prévisionnel | 4 000,00 |
| 20E06174T03 | FEDER - URN - RN-LEGO - Tranche 03 | P270E35-EHN | AP-2015-D-906-FEDER-HN-Souteni | 0,00 | Affectation initiale | Prévisionnel | 36 000,00 |
| | | | | | | ***Sous-Total P270E35-EHN :*** | **511 000,00** |

**Opération P270O004 Projets contractualisés 2014 2020**

| Code Ttranche | Description Tranche | Code AP/EPCP | Libellé AP/EPCP | Mt Pluri affect. | N° Mvt Tranche | Statut Mvt Tranche | Montant Mvt Tranche |
|---|---|---|---|---|---|---|---|
| 20E06142T09 | REGION/CPER- UNICAEN - ARCHADE CHOxTRaCC - Tranche 09 | 270E08-CPE | AE-2015-D-939-CPER-Soutenir la | 0,00 | Affectation initiale | Prévisionnel | 745 834,00 |
| | | | | | | ***Sous-Total P270E08-CPER :*** | **745 834,00** |

| Code Ttranche | Description Tranche | Code AP/EPCP | Libellé AP/EPCP | Mt Pluri affect. | N° Mvt Tranche | Statut Mvt Tranche | Montant Mvt Tranche |
|---|---|---|---|---|---|---|---|
| 20E06142T08 | REGION/CPER- UNICAEN - ARCHADE CHOxTRaCC - Tranche 08 | 270E17-CPE | AP-2015-D-909-CPER-Soutenir la | 0,00 | Affectation initiale | Prévisionnel | 300 000,00 |
| | | | | | | ***Sous-Total P270E17-CPER :*** | **300 000,00** |

**DOSSIERS D'AIDE PRESENTES A LA**

Annexe 2

## Commission permanente du 16 Novembre 2020

| Bénéficiaire | Description de l'opération | Procédure | Montant subventionnable HT ou TTC | Taux aide | Type de subvention | Montant aide | Dates prévisionnelles début et fin de l'opération | Dates de début et de fin d'éligibilité des dépenses | Date limite de transmission des pièces justificatives | Date de fin de convention | code du dossier |
|---|---|---|---|---|---|---|---|---|---|---|---|
| UNIVERSITE DE CAEN NORMANDIE | RIN Recherche 2020 - projet Tremplin - ARCHADE CHOxTRaCC | CPER Investissement | 1 045 834,00 € HT | 100,00% | Proportionnelle | 1 045 834,00 € | Du 01/01/2021 au 31/12/2022 | Du 01/01/2021 au 31/12/2022 | 30/04/2023 | 30/10/2023 | 20E06142 |
| AEP AGENCE NATIONALE SECURITE SANITAIRE ALIMENTATION TRAVAIL | RIN Recherche 2020 - Projet tremplin - AVEq-ACTIVE : Anti-Viraux Equins - Acellular Therapeutic Innovation for Equine | FEDER - Soutien aux projets de recherche (BN) | 102 500,00 € HT | 100,00% | Proportionnelle | 102 500,00 € | Du 01/12/2020 au 30/11/2022 | Du 01/12/2020 au 30/11/2022 | 31/03/2023 | 30/11/2024 | 20E05909 |
| GIP LABEO | | FEDER - Soutien aux projets de recherche (BN) | 160 500,00 € HT | 100,00% | Proportionnelle | 160 500,00 € | Du 01/12/2020 au 30/11/2022 | Du 01/12/2020 au 30/11/2022 | 31/03/2023 | 30/11/2024 | 20E05904 |
| UNIVERSITE DE CAEN NORMANDIE | RIN Recherche 2020 - Projet Tremplin - DYNET : Dynamic Network | FEDER - Soutien aux projets de recherche (BN) | 114 000,00 € HT | 100,00% | Proportionnelle | 114 000,00 € | Du 01/01/2021 au 31/12/2022 | Du 01/01/2021 au 31/12/2022 | 30/04/2023 | 31/12/2024 | 20E06086 |
| UNIVERSITE LE HAVRE NORMANDIE | | FEDER - Soutien aux projets de recherche (HN) | 114 000,00 € HT | 100,00% | Proportionnelle | 114 000,00 € | Du 01/01/2021 au 31/12/2022 | Du 01/01/2021 au 31/12/2022 | 30/04/2023 | 31/12/2024 | 20E06110 |
| INSTITUT NATIO SCIENCES APPLIQUEES ROUEN | RIN Recherche 2020 - Projet tremplin - EFLUX : Développements de nouveaux procédés d'électrosynthèse en flux continu pour l'accès à des architectures moléculaires d'intérêts | FEDER - Soutien aux projets de recherche (HN) | 436 260,00 € HT | 100,00% | Proportionnelle | 436 260,00 € | Du 01/12/2020 au 30/11/2022 | Du 01/12/2020 au 30/11/2022 | 31/03/2023 | 30/11/2024 | 20E05832 |
| ESIGELEC | RIN Recherche 2020 - Projet Tremplin - ETHNOTEVE : Evaluation Thermique de Nouvelles Technologies de puissance dédiées à des applications Véhicules Electriques | FEDER - Soutien aux projets de recherche (HN) | 142 500,00 € HT | 100,00% | Proportionnelle | 142 500,00 € | Du 01/01/2021 au 31/12/2022 | Du 01/01/2021 au 31/12/2022 | 30/04/2023 | 31/12/2024 | 20E05972 |
| UNIVERSITE DE CAEN NORMANDIE | | FEDER - Soutien aux projets de recherche (BN) | 255 000,00 € HT | 100,00% | Proportionnelle | 255 000,00 € | Du 01/01/2021 au 31/12/2022 | Du 01/01/2021 au 31/12/2022 | 30/04/2023 | 31/12/2024 | 20E06084 |

| Bénéficiaire | Description de l'opération | Procédure | Montant subventionnable HT ou TTC | Taux aide | Type de subvention | Montant aide | Dates prévisionnelles début et fin de l'opération | Dates de début et de fin d'éligibilité des dépenses | Date limite de transmission des pièces justificatives | Date de fin de convention | code du dossier |
|---|---|---|---|---|---|---|---|---|---|---|---|
| UNIVERSITE DE CAEN NORMANDIE | RIN Recherche 2020 - Projet Tremplin - EYETRACKING : Suivi automatique du point de regard chez l'humain | FEDER - Soutien aux projets de recherche (BN) | 80 500,00 € HT | 100,00% | Proportionnelle | 80 500,00 € | Du 01/01/2021 au 31/12/2022 | Du 01/01/2021 au 31/12/2022 | 30/04/2023 | 31/12/2024 | 20E06082 |
| UNIVERSITE DE ROUEN-NORMANDIE | | FEDER - Soutien aux projets de recherche (HN) | 108 500,00 € HT | 100,00% | Proportionnelle | 108 500,00 € | Du 01/01/2021 au 31/12/2022 | Du 01/01/2021 au 31/12/2022 | 30/04/2023 | 31/12/2024 | 20E05935 |
| UNIVERSITE DE CAEN NORMANDIE | RIN Recherche 2020 - Projet tremplin - LEGO : « Organ on chip » Like Experimental Global Observations of bacterial biofilms | FEDER - Soutien aux projets de recherche (BN) | 122 500,00 € HT | 100,00% | Proportionnelle | 122 500,00 € | Du 01/01/2021 au 31/12/2022 | Du 01/01/2021 au 31/12/2022 | 30/04/2023 | 31/12/2024 | 20E06170 |
| UNIVERSITE DE ROUEN-NORMANDIE | | FEDER - Soutien aux projets de recherche (HN) | 348 000,00 € HT | 100,00% | Proportionnelle | 348 000,00 € | Du 01/01/2021 au 31/12/2022 | Du 01/01/2021 au 31/12/2022 | 30/04/2023 | 31/12/2024 | 20E06174 |
| PRAXENS | | Soutien aux projets de recherche | 25 000,00 € HT | 100,00% | Proportionnelle | 25 000,00 € | Du 01/01/2021 au 31/12/2022 | Du 01/01/2021 au 31/12/2022 | 30/04/2023 | 31/12/2023 | 20E06177 |
| EPA ECOLE NATIONALE VETERINAIRES D'ALFORT | RIN Recherche 2020 - Projet tremplin - OA-ACTIVE : OsteoArticular Acellular Therapeutic InnoVations for Equine | FEDER - Soutien aux projets de recherche (BN) | 97 450,00 € HT | 100,00% | Proportionnelle | 97 450,00 € | Du 01/01/2021 au 31/12/2022 | Du 01/01/2021 au 31/12/2022 | 30/04/2023 | 31/12/2024 | 20E06128 |
| UNIVERSITE DE CAEN NORMANDIE | | FEDER - Soutien aux projets de recherche (BN) | 202 000,00 € HT | 100,00% | Proportionnelle | 202 000,00 € | Du 01/01/2021 au 31/12/2022 | Du 01/01/2021 au 31/12/2022 | 30/04/2023 | 31/12/2024 | 20E06163 |
| ECOLE NATIONALE SUPERIEURE INGENIEURS | RIN Recherche 2020 - projet Tremplin - PredicAlert | FEDER - Soutien aux projets de recherche (BN) | 61 500,00 € HT | 100,00% | Proportionnelle | 61 500,00 € | Du 01/01/2021 au 31/12/2022 | Du 01/01/2021 au 31/12/2022 | 30/04/2023 | 31/12/2024 | 20E06089 |
| UNIVERSITE DE CAEN NORMANDIE | | FEDER - Soutien aux projets de recherche (BN) | 328 600,00 € HT | 100,00% | Proportionnelle | 328 600,00 € | Du 01/01/2021 au 31/12/2022 | Du 01/01/2021 au 31/12/2022 | 30/04/2023 | 31/12/2024 | 20E06088 |
| UNIVERSITE DE CAEN NORMANDIE | RIN Recherche 2020 - Projet Tremplin - SCHISM : Supporting chemoinformatics via interactive unsupervised and semi-supervised data | FEDER - Soutien aux projets de recherche (BN) | 118 000,00 € HT | 100,00% | Proportionnelle | 118 000,00 € | Du 01/01/2021 au 31/12/2022 | Du 01/01/2021 au 31/12/2022 | 30/04/2023 | 31/12/2024 | 20E06083 |
| UNIVERSITE DE ROUEN-NORMANDIE | | FEDER - Soutien aux projets de recherche (HN) | 116 000,00 € HT | 100,00% | Proportionnelle | 116 000,00 € | Du 01/01/2021 au 31/12/2022 | Du 01/01/2021 au 31/12/2022 | 30/04/2023 | 31/12/2024 | 20E05838 |

| Bénéficiaire | Description de l'opération | Procédure | Montant subventionnable HT ou TTC | Taux aide | Type de subvention | Montant aide | Dates prévisionnelles début et fin de l'opération | Dates de début et de fin d'éligibilité des dépenses | Date limite de transmission des pièces justificatives | Date de fin de convention | code du dossier |
|---|---|---|---|---|---|---|---|---|---|---|---|
| CENTRE NATIONAL DE LA RECHERCHE SCIENTIFIQUE | RIN Recherche 2020 - Tremplin - HAITY : Source THz monocycliques de haute cadence pour la microscopie 4D et la spectroscopie | FEDER - Soutien aux projets de recherche (HN) | 495 000,00 € HT | 100,00% | Proportionnelle | 495 000,00 € | Du 01/12/2020 au 30/11/2022 | Du 01/12/2020 au 30/11/2022 | 31/03/2023 | 30/11/2024 | 20E02190 |
| UNIVERSITE DE CAEN NORMANDIE | RIN Recherche 2020 - Tremplin - Projet HAIS (Human Adaptibility In-Situ) | FEDER - Soutien aux projets de recherche (BN) | 652 651,00 € HT | 100,00% | Proportionnelle | 652 651,00 € | Du 01/01/2021 au 31/12/2022 | Du 01/01/2021 au 31/12/2022 | 30/04/2023 | 31/12/2024 | 20E06087 |
| CENTRE NATIONAL DE LA RECHERCHE SCIENTIFIQUE | RIN Recherche 2020 - Tremplin - SO SIRIUS : Start Of SIRIUS | FEDER - Soutien aux projets de recherche (BN) | 144 000,00 € HT | 100,00% | Proportionnelle | 144 000,00 € | Du 01/01/2021 au 31/12/2022 | Du 01/01/2021 au 31/12/2022 | 30/04/2023 | 31/12/2024 | 20E05189 |
| **TOTAL** | | | | | | **5 270 295,00 €** | | | | | |

# P270 – projets RIN Recherche « Tremplins » - CP du 16 novembre 2020

**Définition d'un projet tremplin**

Soutien à des projets de recherche à fort potentiel et à haut niveau d'ambition scientifique, projetant d'aboutir à une labellisation d'excellence, à une collaboration internationale ou permettant une valorisation socio-économique.
Le dossier de candidature doit faire clairement mention de la labellisation, de l'appel à projet européen/national, de la collaboration internationale ou de la valorisation socio-économique visés à l'issue du projet.

**Dépenses éligibles au titre du règlement 2020**

Frais de personnels non permanents, frais de mission, consommables, équipements, mise à niveau d'équipements, prestations de recherche, aide au montage de dossiers

Ci-dessous, présentation des projets par Réseau d'Intérêt Normand.

- **RIN Normandie Biomédicale et Chimie (NBC) - Pôle ComUE Chimie et biologie appliquées à la santé et au bien-être (CBSB)**

  - **Projet HAIS : Humain Adaptivity in-situ**

Porté par l'Université de Caen Normandie

Le projet consiste à mesurer les capacités d'adaptations physiologiques de l'humain en immersion dans différents environnements extrêmes pour des applications et retombées grand public, sportives, scientifiques, biotechnologiques et médicales. Ce projet propose deux protocoles in-situ dans lesquels des paramètres physiologiques et chronobiologiques seront étudiés dans deux conditions différentes, la première pour évaluer l'impact du climat et de l'ultra-endurance et la seconde pour évaluer l'impact de l'ultra-endurance sur les performances humaines.

  - **Projet PredicAlert**

Porté par l'Université de Caen Normandie et l'ENSICAEN

Le projet vise à développer une nouvelle méthodologie afin de prédire l'état de vigilance à partir de variables cardiorespiratoires utilisables dans des environnements où la technique de référence n'est pas envisageable. Dans le cadre de ce projet, le domaine d'application visé est l'IRM et notamment les études d'IRM fonctionnelle de repos mais d'autres domaines comme le contrôle de la vigilance dans les transports seront possibles.

  - **Projet Archade CHOxTRaCC**

Porté par l'Université de Caen Normandie

Le projet vise à approfondir une des thématiques structurantes de ce projet : le développement de traitements combinés avec l'hadronthérapie pour renforcer son efficacité vis-à-vis des tumeurs radiorésistantes. Il s'agit plus précisément d'explorer l'impact de l'hypoxie tumorale et des conséquences du stress oxydant, lié ou non à ces conditions hypoxiques, sur les cellules tumorales, notamment les cellules souches cancéreuses, et leur environnement extra-cellulaire tumoral ou non.

  - **Projet OA-ACTIVE**

Porté par l'Université de Caen Normandie et l'ENVA

Le projet vise à développer une stratégie thérapeutique acellulaire des arthropathies dans un but de régénération et de protection du cartilage au long court. Deux solutions thérapeutiques intra-articulaires seront développées et évaluées.

- **Projet AVEQ-ACTIVE**

Porté par l'ANSES et LABEO

Ce projet propose donc, en plus des vaccins déjà disponibles pour l'HVE-1 et de l'AVE, d'élargir la panoplie des traitements disponibles pour lutter contre ces virus équins et ainsi limiter les conséquences de ces infections sur la filière équine.

- **Projet EFLUX**

Porté par l'INSA de Rouen Normandie

L'objectif du projet est de créer un centre d'expertise dans le domaine de l'électrosynthèse organique en flux continu à l'échelle de la Normandie à travers l'établissement d'une collaboration internationale avec deux équipes de recherche de l'Université de Montréal qui sont spécialistes dans la synthèse en flux continue. Les complémentarités des expertises des deux partenaires (laboratoire COBRA et UdM) conduiront à la création d'un laboratoire international associé.

- **Projet LEGO**

Porté par les Universités de Caen et Rouen Normandie, Praxens et N2S

L'objectif principal de ce projet est d'étudier in vitro l'effet direct et indirect de facteurs de stress exogènes et endogènes sur la physiologie et le métabolisme de bactéries du microbiote cutané dans un contexte se rapprochant des conditions in vivo.

➢ **RIN Normandie Energies et Matériaux (NEM) - Pôle ComUE Énergies, Propulsion, Matière, Matériaux (EP2M)**

- **Projet HAITY : Source THz monocycliques de haute cadence pour la microscopie 4D et la spectroscopie**

Porté par la Délégation du CNRS de Normandie

Le projet a pour but de de développer une nouvelle génération de microscope 4D pour une large gamme de matériaux. Il apportera des avancées scientifiques majeures dans le domaine des sources THz intenses et permettra des applications scientifiques et industrielles innovantes.

- **Projet SO SIRIUS : Start Of SIRIUS**

Porté par la Délégation du CNRS de Normandie

L'objectif du projet est de valider le fonctionnement de SIRIUS (Selection and Identification of Rare Isotopes Using $S^3$), de quantifier ses performances et de livrer un détecteur fonctionnel pour les

expériences portant sur l'étude de la structure des noyaux superlourds. Ce détecteur sera ensuite mis en œuvre dans de nombreuses collaborations internationales entreprises par le GANIL

- **RIN Normandie Digitale (ND) – Pôle COMUE Sciences du Numérique**

  - **Projet ETHNOTEVE : Evaluation Thermique de Nouvelles Technologies de puissance dédiées à des applications Véhicules Electriques**

Porté par l'Université de Caen Normandie et l'ESIGELEC

Le projet a pour objectif la caractérisation thermique en fonctionnement, c'est-à-dire en régime de commutation de composants de puissance de la filière nitrure de gallium dédiés à des applications véhicules électriques et hybrides.

  - **Projet EYETRACKING : Suivi automatique du point de regard chez l'humain**

Porté par l'Université de Caen Normandie et l'Université de Rouen Normandie

L'objectif du projet est de re-contextualiser le point de regard dans son environnement tridimensionnel à partir des données du masque optique lors d'une tâche d'escalade sur un plan vertical.

  - **Projet DYNET : Dynamic Network**

Porté par l'Université de Caen Normandie et l'Université le Havre Normandie

Le projet vise à développer une méthodologie d'analyse de graphes dynamiques, outils permettant de modéliser l'évolution d'un espace d'états discrets et leurs relations.

  - **Projet SCHISM : Supporting chemoinformatics via interactive unsupervised and semi-supervised data**

Porté par l'Université de Caen Normandie et l'Université de Rouen Normandie

Le projet propose d'explorer une nouvelle approche pour la fouille interactive des données, dans un contexte interdisciplinaire, en collaboration avec des biochimistes et des chémoinformaticiens.
