# Supplementary material for: Study of the Kinetics of the Determinants of Performance During a Mountain Ultramarathon: Multidisciplinary Protocol of the First Trail Scientifique de Clécy 2021
Source: JMIR Res Protoc. 2022 Jun 15;11(6):e38027. doi: 10.2196/38027 (PMC9244647; doi:10.2196/38027)
Supplement: Multimedia Appendix 2 [file resprot_v11i6e38027_app2.docx]

| 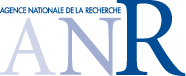 | **Appel à Projets**  NOR-Tremplin | **Edition 2020** |
| --- | --- | --- |
|  | **Rapport de l'expert** |  |

| **Projet** | |
| --- | --- |
| **Acronyme** | **hais** |
| **Titre en français** | **Humain Adaptivity in-situ** |
| **Titre en anglais** |  |
| **Instrument financier** | **Autres AAP spécifiques** |

| **Coordinateur du projet** | | | |
| --- | --- | --- | --- |
| **Prénom** |  | **Nom** |  |
| **Email** | benoit.mauvieux@unicaen.fr | | |
| **Organisme** |  | | |

| **Clarté des objectifs et des hypothèses de recherche** | |
| --- | --- |
|  |  |
| Le consortium formé par Mr Benoit Mauvieux (14 personnes statutaires impliquées, soit 6,1 temps plein sur 18 mois issues de 5 équipes : 4 françaises, 1 suisse, 1 nord-américaine) vise à comprendre les mécanismes d’adaptation du corps humain lors d’activités physiques intenses et/ou soutenues longuement dans différents environnements et/ou contextes de pratique d’activité physique fortement contraignants (climats qualifiés d’extrêmes, et/ou perturbation majeure du sommeil et des rythmes chronobiologiques par ex. par le jet lag).    Les objectifs scientifiques sont clairs et les hypothèses pertinentes. Ils se focalisent sur l’amélioration des connaissances sur les effets de ces pratiques très spécifiques sur :1/ la désynchronisation/resynchronisation des rythmes circadiens physiologiques, 2/ les processus inflammatoires musculaires et systémiques, 3/ les processus cognitifs et psychologiques (mémoire, motivation, prise de décision, fatigue...). |  |

| **Caractère novateur, originalité, positionnement par rapport à l’état de l’art** | |
| --- | --- |
|  |  |
| La réalisation de projets scientifiques en environnements extrêmes (ou agressifs au sens du code du travail), comme celui proposé par Mr Benoit Mauvieux, me parait indispensable pour déterminer les limites des capacités humaines d’adaptation aux divers stress induits (cumulés) par de telles conditions de pratiques sportives ET de travail (cf. domaine de l’« occupational health/safety » juste évoqué dans le projet : ex. sapeurs-pompiers en intervention feu et militaires en OPEX).    L’intérêt fondamental du présent projet est de se placer délibérément en situation écologique et non en situation de simulation de l’environnement en laboratoire (deux approches complémentaires dont la seconde est généralement plus développée car plus aisée, moins coûteuse, permettant l’utilisation de matériel intransportable, … ). Par ailleurs, l’idée est de mesurer (par une batterie de tests biologiques et psychologiques) et non simplement rapporter des observations personnelles (ex. cahier d’observation).  La condition qualifiée de in situ dans le projet impose le montage d’expéditions en terrains extrêmes (i.e. 4x30 mission 20 de l’Human Adaptation Institute / dir. Christian Clot) ou d’évènements sportifs (i.e. UTSPC) impliquant un nombre de sujets suffisants (/contraintes statistiques, 20 pour la partie 4x30 mission 20 et 50 pour l’UTSPC) ET le développement d’outils/d’équipements de mesure des paramètres physiologiques résistants à ces environnements agressifs et aisément transportable. Des partenaires industriels sont impliqués dans ce dernier point.  Le caractère original du projet réside dans le recueil de données en conditions per- évènements (outre les classiques conditions pré- et post-évènements) permettant d’établir une véritable cinétique sur les variables physiologies et cognitives d’intérêt.    Sans que cela soit rédhibitoire sur ma perception de l’intérêt du présent projet, je dois signaler que contrairement à la vision avancée par Mr Benoit Mauvieux, des études tant physiologiques que psychologiques bien antérieures (certes avec des équipements moins modernes) ont déjà été réalisées in situ dans nombre d’environnements extrêmes (certes différents de ceux ciblés ici : ainsi beaucoup de travaux portent sur himalayisme et très hautes altitudes, plongée à saturation/COMEX, Espace, stations antarctiques, …).  Voir par ex. le volumineux « Medical Aspects Of Harsh Environments Volume 1 et 2 », by U.S. Army. Office of The Surgeon General. Borden Institute pour ce qui est des environnements extrêmes, lieux d’exercice des militaires. Dès lors, il pourrait être judicieux de se rapprocher de l’IRBA, très au fait de ce genre d’études et surtout de leurs implications pour les Forces armées dans le cadre d’opérations en milieux extrêmes, ou de missions à forte privation de sommeil.    Enfin, et assez curieusement, la notion (certes ancienne) d’adaptation croisée n’apparait pas dans la partie dédiée à l’évènement 4x30 – mission 20 alors qu’un renouveau sur ce type d’effets croisés se fait jour en physiologie humaine (e.g. adaptation croisée « milieu chaud /milieu hypoxique » sur le développement du VO2 max). Cela est étonnant dans la mesure ou les 20 participants enchaîneront les séjours dans les environnements extrêmes. On touche là d’ailleurs à l'une des limites du protocole. Ainsi, les 20 sujets seront tous immergés ensemble dans le même environnement alors qu’il aurait été plus judicieux de les y incorporés par groupe plus restreint mais dans un ordre aléatoire. Il n’y a par ailleurs pas de groupe-situation témoin (i.e. les 20 sujets réalisant dans un environnement non agressif, ce qu’ils feront en marche et portage-tirage dans les 4 environnements extrêmes). Cela aurait rajouté 30 jours supplémentaire au (sous)projet.    J’ai conscience que cela n’est que peu réalisable du fait que l’évènement 4x30 -mission 20 n’est ni sous la responsabilité ni le financement de Mr Bruno Mauvieux. En effet, cet évènement-expédition est piloté entièrement par l’IAH (Institute of Human Adaptability) porté par Mr Christian Clot. |  |

| **Pertinence de la méthodologie, gestion des risques scientifiques** | |
| --- | --- |
|  |  |
| Le projet repose sur 11 axes, chacun utilisant des méthodes classiques pour l’étude des variables physiologiques et psychologiques (ou cognitives) d’intérêt. Par ailleurs, chacune des personnes statutaires impliquées dans ce projet maîtrise pleinement les méthodologies qui seront utilisées. Celles-ci sont classiques, et seront « simplement » (avec des adaptations indispensables) transposées des laboratoires vers le terrain (EEG, prélèvements sanguins, actimétries, …).  Concernant les analyses biologiques planifiées dans le cadre de l’évènement 4x30 -mission 20, il me parait étonnant les certains marqueurs suivis soient choisis de manière univoque c’est-à-dire sans tenir compte du milieu. Sans l’espérer, l’exposition de 20 sujets au milieu chaud peut conduire à des troubles voire des pathologies thermiques (e.g. coup de chaleur d’exercice). Dès lors, il serait judicieux de suivre l’évolution des LPS sanguins lors de l’exposition au désert iranien, et à l’inverse celle des cryoglobulines en Sibérie.  Enfin, je n’ai pas clairement perçu que les sujets des 2 évènements (4x30 mission 20 et UTSPC seront « monitorés » en continu par cardiofréquencemétrie permettant une analyse (a posteriori ou temps réel) de la variabilité sinusale, « indicateur classique » de la balance ortho/parasympathique, donc de l’état de fatigue et de la qualité de la récupération (surtout pendant le sommeil).  Les principaux risques scientifiques résident : 1/ dans la coordination d’un grand nombre d’acteurs scientifiques (7 équipes dont 6 sur le terrain en condition « per »), 2/ la nécessité de préserver les grands effectifs de participants aux 2 évènements (50 à parité pour l’UTSPC, et 20 à parité durant plus de 4 mois sur 4 environnements extrêmes pour le 4x30 -mission 20), et enfin et surtout 3/ dans le fait que pour le 4x30 -mission 20, Mr Benoit Mauvieux n’est ni l’organisateur, ni le financeur du (sous)projet. C’est probablement sur ce dernier point que l’aléas est le plus fort : annulation d’une partie, plusieurs parties ou de toute l’expédition organisée par l’IHA.    On remarquera sur ce dernier point que le projet décrit sur le site de l’IHA précise que le premier séjour aura lieu en 2019 (http://www.adaptationexpe.com/fr/la-mission-20-fr/), ce qui serait incompatible avec le déroulé présenté pour l’étude scientifique et donc avec la demande de financement afférente. Ce point devra impérativement être éclairci avant l’obtention du financement. |  |

| **Compétence, expertise et implication du coordinateur scientifique et des partenairese** | |
| --- | --- |
|  |  |
| Le porteur de projet, Mr Benoit Mauvieux, a déjà coordonné des projets scientifiques de même nature (Grand Raid de la Réunion 2017, projets EMPHASE) ce qui démontre sa capacité à conduire des opérations scientifiques de terrain loin de sa base normande.    Toutes les personnes statutaires impliquées dans ce projet sont toutes dotées d’une grande expérience (compétence et expertise) dans leurs domaines respectifs de recherche ; cela est attesté par une liste très significative de publications récentes dans des revues internationales indexées.    Les partenaires potentiels ont marqué leur intérêt au présent projet par des lettres d’intention/soutien (à l’exception du Pr. Grégoire Millet de l’ISSUL-Suisse).  Enfin, il est a signalé que certains participants extérieurs au laboratoire COMETE (porteur du projet) apporteront une contribution financière à la réalisation des études les impliquant (axes 8 et 9, Pr Millet autofinancement ISSUL ; axe 10, Dr Johnson et al, financement de 35 / 50 keuros de leur participation).    La partie analyse cognitive par IRM pré/post 4x30 -mission 20 se fera au LNC Paris (Pr Etienne Koechlin, UMR INSERM ENS U960) ; cette partie est financée indépendamment par un contrat de 300 k euros (ANR ASTRID DGA, 2019-2021). |  |

| **Qualité et complémentarité du consortium, qualité de la collaboration** | |
| --- | --- |
|  |  |
| Le consortium formé par Mr Benoit Mauvieux (14 personnes statutaires impliquées, soit 6,1 temps plein sur 18 mois- issues de 5 équipes : 4 françaises, 1 suisse, 1 nord-américaine) s’inscrit pleinement dans une logique de complémentarité.  Les compétences en physiologie, en psychologie et cognition, médecine et technologie requises pour menées à bien le projet sont toutes représentées par des universitaires (enseignants-chercheurs, médecins hospitaliers, ingénieurs de startups).    A l’évidence, certaines fonctions physiologiques ne seront pas étudiées durant les évènements (UTSPC ou 4x30 mission 20). Cela est très compréhensible et donc acceptable dans le cadre d’une étude de terrain. Par exemple lors de l’UTSPC, certaines mesures et recueil des données en condition « per » ne pourront se faire que durant le passage (8 prévus) à la base de loisirs Lionel Terray, de fait transformée en laboratoire de recherche. La multiplication des tests induirait alors des pauses très (trop) longues pouvant transformer l’ultra trail en une répétition de 8 courses brèves (20 km) dénaturant la course et le projet scientifique. |  |

| **Adéquation des moyens mis en oeuvre et demandés aux objectifs du projet** | |
| --- | --- |
|  |  |
| Du point de vue scientifique, les moyens humains et matériels qui seront mis en œuvre sont conséquents. Des études préliminaires ont été réalisées (EMPHASE sur le Grand Raid de la Réunion en 2017, le Rallye Dakar 2017, …), s’assurant de la faisabilité de certains points expérimentaux dont la fiabilité des équipements de terrain.    Le financement demandé pour 3 années est conséquent : 745,8 keuros dont plus de 99% en demande de subvention à la région Normandie. Un financement de 300 keuros a déjà été obtenu en 2019 (également pour 3 ans ; ANR ASTRID DGA – UMRS INSERM ENS U960) pour l’étude cognitive.    Le financement demandé à la région Normandie porte pour :  - 41% sur la rémunération de doctorants (2), de post-doctorants (2) et d’un ingénieur de recherche sur 6 mois,  - 30% sur le coût de fonctionnement (consommable dont 1200 gélules e-Celsius pour 60 keuros, dosages sanguins = 63 keuros, déplacements = 24 keuros, …),  - 27% sur le coût en équipements (25 brassières Sense = 50 keuros, casque EEG = 30 keuros ; ordinateur de plongée Mares= 7,7 keuros, casque EEG, …).  Si le projet est conduit à son terme selon le déroulé prévu, le budget demandé me semble en adéquation avec les besoins des études (11 axes). |  |

| **Impact scientifique et impact potentiel dans les domaines économique, social ou culturel** | |
| --- | --- |
|  |  |
| Le projet s’inscrit dans la continuité des études conduites par les différents personnels statutaires impliqués dans le consortium construit pour ce projet. Il conduira donc naturellement à la rédaction et publication d’articles scientifiques dans des revues internationales indexées.    Le suivi scientifique de l’évènement 4x30 – mission 20, suite de l’expédition de Christian Clot 4x30 Solo, fera certainement l’objet d’une couverture médiatique importante (comme ce fut le cas pour l'expédition préliminaire en Solo).    Le projet repose sur un consortium majoritairement local associant 4 unités de recherche sur 2 universités normandes, le CHU de Caen, une fondation et des startups régionales. Il ressortira de ce projet fédérateur de compétences la création locale d’une expertise collective sur les études de terrain en environnements extrêmes et sur les activités d’ultra endurance (ultra trail en particulier).    La création de l’ultra trail scientific project de Clécy (UTSPC à répéter sur 3 ans) mettra en valeur la base de loisir Lionel Terray de Clécy, et devrait mener à l’organisation d’un colloque international sur le trail et l’ultra endurance. |  |

| **Projection d’un dépôt de projet dans le cadre d’AAP national ou européen, ou actions de transferts vers le monde socio-économique ou partenariat public-privé envisagés à l'issue du projet** |
| --- |
| Non renseigné dans le document (où je ne l’ai pas trouvé tel quel).    Il est mentionné que la partie analyse IRM du cerveau-cognition des participants de l’évènement 4x30 – mission 20, portée par le Pr. Etienne Koechlin (UMRS INSERM ENS Paris, U960) fait déjà l’objet d’un financement sur 3 ans: ANR ASTRID DGA, 300 k euros, 2019.    Le projet 4x3 mission solo de Christian Clot (sept 2016-2017) a eu un fort impact médiatique. Il est raisonnable d'avancer que si le projet 4x30 - mission 20 va a sort terme, il sera promis à une reconnaissance médiatique plus importante encore, du fait que le nombre de participants permettra de tirer plus d'enseignements sur les capacités adaptatives humaines. |

| **Points forts** | |
| --- | --- |
|  |  |
| Compétence et expertise du personnel statutaire impliqué ;  consortium aux éléments très complémentaires ;  approche expérimentale de terrain (situation écologique dite in situ);  évènements de terrain exceptionnels surtout le 4x30 – mission 20 (4 environnements extrêmes enchaînés pour 20 sujets) ;  développements d’outils technologiques par des startups régionales ;  soutien de l’ANR sur une partie du projet, soutien de Fondation ;  faisabilité / situations de travail in situ testées en 2017 au Grand Raid de la Réunion (EMPHASE)  retombées économiques locales pour l'évènement (sous projet) UTSPC  retombées médiatiques importantes pour l'évènement (sous projet) 4x30 - mission 20    40% du financement demandé à la Région Normandie servira à rémunérer 2 thésards, 2 post-doctorants et un IgR |  |

| **Points faibles** | |
| --- | --- |
|  |  |
| 1/ L’évènement de terrain 4x30 – mission 20 (4 environnements extrêmes enchaînés pour 20 sujets) échappe complètement au contrôle logistique et organisationnel du porteur de projet scientifique. Son annulation rendrait caduque le projet scientifique et la demande de financement afférente.    2/ Le projet scientifique repose sur la réalisation de 2 évènements (UTSP Clécy et 4x30 – mission 20) qui ont peu de choses en commun. Cela ressemble plus à une juxtaposition de 2 projets scientifiques reposant sur des compétences communes qu’à un projet unique visant un but scientifique cohérent sur ses 2 branches (i.e. ultra trail et sportifs accomplis vs environnements extrêmes et des citadins non sportifs).    Globalement, la demande de financement pourrait ne porter que le soutien des études concernant l’un ou l’autre des 2 évènements. Dans cet esprit, le projet 4x30 – mission 20 me parait plus intéressant scientifiquement bien que moins abouti (pas de situation témoin) alors que le projet UTSPC me parait plus abouti, plus porteur de retombées locales, mais en contrepartie de portée moins générale (intérêt limité à l’univers de l’ultra endurance qui est un plein essor ). |  |

| **Synthèse** |
| --- |
| Le projet porté par Mr Benoit Mauvieux vise à mieux comprendre les mécanismes adaptatifs (psychologiques et cognitif, et physiologiques) de l'humain placé en situation de fort stress pour des périodes longues.Ce stress est induit par : 1/ un travail physique soutenu (trail) entre 20 et 30 heures impliquant une privation de sommeil et donc une forte perturbation des rythmes biologiques ou 2/ des marches avec portage ou tirer-rouler de charges lourdes dans des environnements naturels extrêmes.    Le point majeur réside dans le fait de l'organisation d'un suivi de nombreuses variables d'intérêt psychologiques, physiologiques et cognitives interrogeant l'impact sur la santé (biopsychosociale) de ces conditions de pratiques physiques extrêmes (ou agressives au sens du code du travail). Il est clair que l'approche globale de l'étude de l'humain dans ce tels milieux ou contexte de pratique a été oubliée durant des décennies privilégiant soit des approches en laboratoire soit des approches de terrain mais focalisant sur des approches très disciplinaires (physiologique OU psychologique).  Dans cet esprit, ce projet renoue avec des approches plus intégratives comme ce fut le cas pour l'étude de l'himalayisme ou de la plongée profonde ou la conquête spatiale. Cela est évidement très prometteur scientifiquement. Je soutiens donc sans réserve (scientifique) le financement de ce projet. |
