# Supplementary material for: Study of the Kinetics of the Determinants of Performance During a Mountain Ultramarathon: Multidisciplinary Protocol of the First Trail Scientifique de Clécy 2021
Source: JMIR Res Protoc. 2022 Jun 15;11(6):e38027. doi: 10.2196/38027 (PMC9244647; doi:10.2196/38027)
Supplement: Multimedia Appendix 3 [file resprot_v11i6e38027_app3.docx]

| 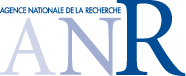 | **Appel à Projets**  NOR-Tremplin | **Edition 2020** |
| --- | --- | --- |
|  | **Rapport de l'expert** |  |

| **Projet** | |
| --- | --- |
| **Acronyme** | **hais** |
| **Titre en français** | **Humain Adaptivity in-situ** |
| **Titre en anglais** |  |
| **Instrument financier** | **Autres AAP spécifiques** |

| **Coordinateur du projet** | | | |
| --- | --- | --- | --- |
| **Prénom** |  | **Nom** |  |
| **Email** | benoit.mauvieux@unicaen.fr | | |
| **Organisme** |  | | |

| **Clarté des objectifs et des hypothèses de recherche** | |
| --- | --- |
|  |  |
| Les objectifs du projet HAIS a pour objectif principal d'étudier l’adaptabilité de l'homme "in situ" en conditions réelles  Il se compose de deux projets distincts  - un projet d'étude de l'adaptation de l'homme (20 personnes à chaque fois) à 4 environnements extrêmes différents  - un projet d'étude de la cinétique d'adaptation de l'homme à un stimulus extrême (une course d'ultra-trail) : le projet UTSPC    Les objectifs affichés sont clairs. |  |

| **Caractère novateur, originalité, positionnement par rapport à l’état de l’art** | |
| --- | --- |
|  |  |
| Les deux projets sont très innovants par leur approche méthodologiques, l'utilisation de matériel embarqué permettant le recueil de données en conditions de vie réelles sont des apports majeurs pour l'étude de l'adaptation de l'homme à différentes conditions ou différents stimuli.    Les réponses potentiellement obtenues à partir des deux projets sont susceptibles d'apporter des réponses à des questions scientifiques dans les différents domaines explorés. Ils sont donc parfaitement positionnés. |  |

| **Pertinence de la méthodologie, gestion des risques scientifiques** | |
| --- | --- |
|  |  |
| Les risques sont bien maîtrisés même si certains ne peuvent être anticipés mais sont inhérents aux études de terrain.    J'ai quelques remarques sur le plan méthodologique.  Projet conditions extrêmes : s'agit-il des mêmes 20 sujets qui vont faire les 4 expéditions ? si oui combien  Les paramètres recueillis sont à la fois des mesures physiologiques, des questionnaires et des mesures sanguines à différents points - il s'agit d'une étude de chronobiologie. Les sujets ont aussi des IRM cérébrales avant le départ et au retour. Les modifications observées pourraient potentiellement être importantes ; aussi n'y aurait-il pas à étudier la "réversibilité" des modifications qui sont aussi des caractères importants de l'adaptabilité  Concernant, les paramètres enregistrées, il est dommage de ne pas voir apparaître des paramètres "vitaux" facile à recueillir et non chronophage (la pression artérielle, la Fc, l'étude de la balance sympato-vagale etc...)    Projet UTSPC : il s'agit d'étudier un groupe de trailer sur une course formatée spécialement pour l'étude en course des adaptations. La course est constituée de plusieurs boucles avec un passage obligé par un laboratoire tous les 20 km . Onze axes, portés par des équipes reconnues, balayent beaucoup de champs physiologiques. il es compliqué à la lecture des différents axes de comprendre s'il s'agit du même groupe de coureurs qui bénéficiera des 11 axes ou si les 11 axes sont différents et autonomes.  Dans le premier cas, le risque est que les coureurs se lassent des différents paramètres à recueillir ; il est du coup dommage car le temps de recueil éloignera ces coureurs de la vraie vie de l'ultra-trailer. Dans le second cas, sans doute plus gérables sur le plan pratique, il y aura une perte d'informations et de liens entre les différents paramètres.  ces points nécessitent d'être éclaircis. |  |

| **Compétence, expertise et implication du coordinateur scientifique et des partenairese** | |
| --- | --- |
|  |  |
| Les équipes sont reconnues et compétentes dans leurs domaines d'expertise. |  |

| **Qualité et complémentarité du consortium, qualité de la collaboration** | |
| --- | --- |
|  |  |
| De nombreuses collaborations scientifiques ont été développée au niveau Européen et international. |  |

| **Adéquation des moyens mis en oeuvre et demandés aux objectifs du projet** | |
| --- | --- |
|  |  |
| Les moyens demandés sont en adéquations avec les hypothèses de travail et les objectifs du projet. |  |

| **Impact scientifique et impact potentiel dans les domaines économique, social ou culturel** | |
| --- | --- |
|  |  |
| Les impacts scientifiques sont réels et ne peuvent pas être mis en cause, à la fois en terme de publications de haut niveau, de communications à des congrès nationaux et internationaux, de retombées médiatiques ou d'ouvrage scientifiques.    Dans le domaine économique, des telles études ne peuvent qu'avoir des retombées positives pour les entreprises locales engagées dans le projet (validation de capteurs embarqués de mesure dans différentes conditions) et la réalisation de congrès régionaux (un congrès est prévu à Caen).    Sur le plan socio-culturelle, les résultats attendus devraient permettre d'informer et peut-être de modifier les comportements ou d'influencer des décisions. |  |

| **Projection d’un dépôt de projet dans le cadre d’AAP national ou européen, ou actions de transferts vers le monde socio-économique ou partenariat public-privé envisagés à l'issue du projet** | |
| --- | --- |
|  |  |
| Ce projet est susceptible, par son caractère innovant d'être soutenu à un AAP national ou européen. |  |

| **Points forts** | |
| --- | --- |
|  |  |
| Le caractère innovant et la solidité des équipes scientifiques. |  |

| **Points faibles** | |
| --- | --- |
|  |  |
| La méthodologie gagnerait à être affinée et précisée (remarques plus hauts) |  |

| **Synthèse** | |
| --- | --- |
|  |  |
|  |  |
