# Supplementary material for: Study of the Kinetics of the Determinants of Performance During a Mountain Ultramarathon: Multidisciplinary Protocol of the First Trail Scientifique de Clécy 2021
Source: JMIR Res Protoc. 2022 Jun 15;11(6):e38027. doi: 10.2196/38027 (PMC9244647; doi:10.2196/38027)
Supplement: Multimedia Appendix 4 [file resprot_v11i6e38027_app4.docx]

| 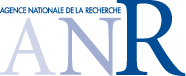 | **Appel à Projets**  NOR-Tremplin | **Edition 2020** |
| --- | --- | --- |
|  | **Rapport de l'expert** |  |

| **Projet** | |
| --- | --- |
| **Acronyme** | **hais** |
| **Titre en français** | **Humain Adaptivity in-situ** |
| **Titre en anglais** |  |
| **Instrument financier** | **Autres AAP spécifiques** |

| **Coordinateur du projet** | | | |
| --- | --- | --- | --- |
| **Prénom** |  | **Nom** |  |
| **Email** | benoit.mauvieux@unicaen.fr | | |
| **Organisme** |  | | |

| **Clarté des objectifs et des hypothèses de recherche** | |
| --- | --- |
|  |  |
| HAIS est un projet original, complémentaire au projet ANR ASTRID –DGA- « Neuro-adapt », dont la finalité est l’étude des capacités d’adaptations psycho-physiologiques humaines en conditions extrêmes. Ce projet qui se décline sous la forme de 2 études (4x30 jour et UTSPC) tente de répondre à une problématique d’actualité, à savoir, quelles sont les capacités d’adaptations humaines au regard de l’évolution des conditions climatiques et des contraintes psycho-physiologiques et physiques auxquelles l’homme devra faire face dans les décennies à venir. Les hypothèses avancées reposent sur les capacités 1) de synchronisation des rythmes biologiques des principales fonctions dans ces environnements comme une réponse d’adaptation et de résonnance écologique 2) d’adaptation psycho-physiologiques et biomécaniques humaines lors d’un exercice de très longue durée. |  |

| **Caractère novateur, originalité, positionnement par rapport à l’état de l’art** | |
| --- | --- |
|  |  |
| La grande originalité de ce projet est d’étudier en conditions écologiques (et non en laboratoire) les mécanismes d’adaptation de l’humain confronté à une combinaison extrême de stress thermique, psychophysiologique et physique. De plus, la réalisation de mesures en continue au cours des épreuves/séjours constitue un positionnement scientifique novateur par rapport à la plupart des précédentes études publiées dans la littérature qui se contentent souvent d’évaluations pré/post observations. Par ailleurs, l’exploration de la plasticité cérébrale en relation avec l’environnement extrême ouvre des perspectives très complémentaires. |  |

| **Pertinence de la méthodologie, gestion des risques scientifiques** | |
| --- | --- |
|  |  |
| La méthodologie utilisée s’appuie sur une approche holistique associant plusieurs champs disciplinaires (physiologie, biomécanique, sciences cognitives, neurosciences, psychologie). Cette approche plurielle au travers d’outils et de méthodes d’investigation variées, complémentaires et innovantes devrait apporter des informations précieuses répondant aux hypothèses de l’étude.  La gestion des risques scientifiques a été abordée de manière réaliste avec plusieurs études exploratoires réalisées soit sur un effectif restreint et/ou dans des conditions expérimentales très proches. Cette phase exploratoire a permis de mettre à jour certaines limites ou dysfonctionnements potentiels qui pourront être corrigés lors du lancement de l’étude à grande échelle. L’ensemble de ces précautions et ajustements devraient limiter les risques de mauvais déroulement des expérimentations. |  |

| **Compétence, expertise et implication du coordinateur scientifique et des partenairese** | |
| --- | --- |
|  |  |
| Le porteur du projet, Benoit Mauvieux, fait état d’une bonne expérience dans l’étude des adaptations humaines en milieu extrême. Il s’est entouré d’une équipe de collaborateurs dont l’expertise dans leur domaine de recherche est incontestable aux yeux de la communauté scientifique internationale. Les partenaires privés (CSEM, SportOnSciences, Bodycap, …) devraient apporter des solutions innovantes pour lever les verrous technologiques identifiés. |  |

| **Qualité et complémentarité du consortium, qualité de la collaboration** | |
| --- | --- |
|  |  |
| Les partenaires institutionnels impliqués dans le projet (COMETE, LAC, EA7369, UMRS INSERM-ENS U960, MAYO Clinic, …) sont en adéquation avec les enjeux scientifiques du projet. |  |

| **Adéquation des moyens mis en oeuvre et demandés aux objectifs du projet** | |
| --- | --- |
|  |  |
| Les moyens mis en œuvre et demandés semblent corrects au regard des montants demandés en terme d’investissement et de fonctionnement pour la réalisation du projet |  |

| **Impact scientifique et impact potentiel dans les domaines économique, social ou culturel** | |
| --- | --- |
|  |  |
| L’originalité et les objectifs du projet HAIS devraient permettre de répondre à un certain nombre de questions relatives aux capacités d’adaptations humaines en milieu extrêmes. Le partenariat avec le secteur privé (par exemple Bodycap) semble une opportunité pour ces entreprises de développer, tester et commercialiser différents dispositifs embarqués et utilisables en milieu extrême avec des applications possibles dans les domaines du sport, de la santé, du travail et des loisirs. |  |

| **Projection d’un dépôt de projet dans le cadre d’AAP national ou européen, ou actions de transferts vers le monde socio-économique ou partenariat public-privé envisagés à l'issue du projet** | |
| --- | --- |
|  |  |
| La création d’un Labcom entre l’Université de Caen et un partenaire privé (par exemple Bodycap) pourrait être un moyen de prolonger l’étude. |  |

| **Points forts** | |
| --- | --- |
|  |  |
| - Projet original répondant aux questionnements actuels sur les capacités d’adaptations humaines dans un environnement en pleine évolution en relation avec des enjeux économiques, sociétaux et de santé publique.  - Approche pluridisciplinaire  - Le porteur et ses collaborateurs sont reconnus pour leurs compétences dans les champs scientifiques impliqués dans l’étude  - Le management des expéditions entièrement indépendant du projet scientifique permet aux chercheurs de se concentrer exclusivement sur leur domaine.  - Existence d’études et missions exploratoires ayant permis de tester la faisabilité des 2 expérimentations |  |

| **Points faibles** | |
| --- | --- |
|  |  |
| La dimension humaine et sociale du projet est un peu sous-estimée. En effet, le protocole 4 x 30 jours va mobiliser une cohorte de 20 individus (10 femmes et 10 hommes, menant par ailleurs une vie citadine occidentale « standard ») qui vont devoir vivre ensemble dans des conditions extrêmes d’isolement et de confinement. L’évolution des relations inter-individuelles, même si cette dimension sera évaluée par la prosocialité, est sans doute un point fondamental qui pourra conditionner la réussite ou l’échec d’une telle expédition. Les investigateurs devront être vigilants sur ce point. L’intégration dans le protocole d’un versant sociologique permettrait d’apporter des informations précieuses qui donneraient encore plus d’envergure au projet |  |

| **Synthèse** | |
| --- | --- |
|  |  |
| Le Projet HAIS (Human Adaptibility In-Situ) a pour but de mesurer les capacités d’adaptations de l’humain en immersion dans différents environnements extrêmes pour des applications et retombées grand public, sportives, scientifiques, biotechnologiques et médicales. Ce projet propose deux protocoles in-situ dans lesquels des paramètres physiologiques et chronobiologiques seront étudiés dans deux conditions différentes, la première (étude 4x30 Jours) pour évaluer l’impact du climat et de l’ultra-endurance et la seconde (étude Ultra Trail Sciences Project Clécy – Suisse Normande) pour évaluer l’impact de l’ultra-endurance sur les performances humaines. Les hypothèses avancées reposent sur les capacités 1) de synchronisation des rythmes biologiques des principales fonctions dans ces environnements comme une réponse d’adaptation et de résonnance écologique 2) d’adaptations psycho-physiologiques et biomécaniques humaines lors d’un exercice de très longue durée. La méthodologie utilisée et les moyens humains, matériels et financiers demandés semblent en adéquations avec les objectifs du projet. L’intégration d’une étude sociologique au protocole actuel apporterait peut-être un éclairage complémentaire pour mieux comprendre les mécanismes d’adaptation de l’homme en situation extrême. Pour conclure, ce projet de recherche pourrait apporter des réponses importantes au regard des enjeux scientifiques, économiques et sociétaux auxquels l’homme va devoir faire face dans les prochaines années. |  |
